# Recent Progress in Non-Enzymatic Electroanalytical Detection of Pesticides Based on the Use of Functional Nanomaterials as Electrode Modifiers

**DOI:** 10.3390/bios12050263

**Published:** 2022-04-20

**Authors:** Tanja Vrabelj, Matjaž Finšgar

**Affiliations:** Faculty of Chemistry and Chemical Engineering, University of Maribor, Smetanova ulica 17, 2000 Maribor, Slovenia; tanja.vrabelj@um.si

**Keywords:** electroanalysis, non-enzymatic sensors, nanomaterials, pesticides

## Abstract

This review presents recent advances in the non-enzymatic electrochemical detection and quantification of pesticides, focusing on the use of nanomaterial-based electrode modifiers and their corresponding analytical response. The use of bare glassy carbon electrodes, carbon paste electrodes, screen-printed electrodes, and other electrodes in this research area is presented. The sensors were modified with single nanomaterials, a binary composite, or triple and multiple nanocomposites applied to the electrodes’ surfaces using various application techniques. Regardless of the type of electrode used and the class of pesticides analysed, carbon-based nanomaterials, metal, and metal oxide nanoparticles are investigated mainly for electrochemical analysis because they have a high surface-to-volume ratio and, thus, a large effective area, high conductivity, and (electro)-chemical stability. This work demonstrates the progress made in recent years in the non-enzymatic electrochemical analysis of pesticides. The need for simultaneous detection of multiple pesticides with high sensitivity, low limit of detection, high precision, and high accuracy remains a challenge in analytical chemistry.

## 1. Introduction

Increasing populations and changes in food consumption patterns have led to growing challenges to intensify agricultural production to satisfy an increasing demand for food and feed and, at the same time, to meet the quality standards commanded in international trade. On the other hand, these facts together lead to increased use of pesticides, which are intended for repelling, destroying, or controlling any pest, regulating the growth of plants, and, nevertheless, also include substances that are applied to crops to protect them from deterioration during storage and transport [1,2,3]. There are many advantages of using pesticides, such as increased food quality and quantity due to crop protection technologies, which allow producers to increase crop yields and the efficiency of food production processes. Many fruits and vegetables would be in short supply if pesticides were not employed, and, consequently, prices would rise. Pesticides are also the most effective substances to eliminate insects that cause human diseases such as Malaria, West Nile virus, etc. Nevertheless, the use of crop protection chemicals in a responsible and safe manner includes household pest control, control of vegetation in industry and infrastructure, recreation, and the protection of areas against environmental pests such as noxious weeds, feral animals, etc, which cause land degradation [4].

However, uncontrolled use of pesticides can lead to water, soil, and air contamination, which transfers the used chemical residues along a food chain and results in changes in natural biological balances, reducing beneficial and nontarget organisms and in the evolution of pesticide resistance in pests [4]. There are reports of high incidences of contamination and poisoning of pesticide users, agricultural workers, and bystanders, and contamination of drinking water resources with pesticides or their breakdown products in many developing countries. The widespread occurrence of residues of certain pesticides in the ground and surface water has, therefore, become a factor in banning or restricting the use of these products due to the risk of increasing long-term health effects, including carcinogenic and endocrine-disrupting properties [3,5].

Pesticide toxicity in living beings results from ingestion, inhalation, or dermal absorption, and continued exposure to these chemicals for an extended period may result in neurological, psychological, and behavioural dysfunctions, hormonal imbalances, immune system dysfunction, reproductive system defects, cancers, genotoxicity, blood disorders and abnormalities in the liver and kidneys, etc. [4]. The most commonly used classes of pesticides (according to their chemical structure) are arsenic compounds, carbamates, nitrophenol derivatives, organochlorine, and organophosphorus compounds. For example, for humans, overuse of arsenic compounds and nitrophenol derivatives can cause stomach ache, nausea, vomiting, or diarrhoea, while carbamates and organophosphorus compounds mainly affect the central nervous system [6]. Although pesticides continue to play an important role in pest management, they also pose significant risks to human health and the environment. Growing concerns about their use appear in various essential sectors, including Health, Environment, Agriculture, and Trade. According to these facts, monitoring and exposure data are critical to determining the impact of pesticides on human health and the environment accurately [7,8].

Pesticides have traditionally been detected using gas chromatography (GC) using different detectors [9,10], gas chromatography-mass spectrometry (GC-MS) [11,12], high-performance liquid chromatography (HPLC) [13,14,15] in association with headspace (HS), liquid-phase microextraction (LPME) [16,17], and solid-phase microextraction (SPME) [13,18]. Although these techniques offer high sensitivity and selectivity with a low limit of detection (LOD), several shortcomings of the conventional methods have restricted their field of applications greatly. The latter include sample decomposition, a limited number of samples in a certain time interval- and matrix interferences, high costs, use of toxic organic reagents, required trained personnel, and unsuitability for real-time detection [15,19,20]

The advancement in miniaturisation and microfabrication technology has led to the development of sensitive and selective electrochemical devices for field-based environmental monitoring of various pollutants. Such devices have found applications in the fields of clinical, industrial, environmental, and agricultural analyses for monitoring water quality parameters, measurements of trace metals in natural water, the presence of carcinogenic compounds and, furthermore, for monitoring organic pollutants such as pesticides in groundwater, tap water, and their presence in food [21,22,23,24,25].

Electrochemical sensors are an important subgroup of chemical sensors, consisting of an electrode-transduction element covered by a recognition layer. On the surface of this layer, the interaction with the target analyte occurs, and the chemical changes resulting from this interaction are translated by the transduction element into electrical signals. Electrochemical sensors are characterised by their small size, cost efficiency, high sensitivity and selectivity, wide linear range, and minimal power consumption. In addition, one of the essential properties for developing and future technologies is the on-site use of such sensors for direct measurement of the analyte in the sample matrix and real-time measurements without the need for sample preparation in the laboratory [21,22].

One of the possible classifications of electrochemical sensing approaches for the detection of pesticides is based on the fact of whether biomolecules are used as recognition elements or not. In this regard, electrochemical sensors can be divided into four main groups: immunosensors, enzyme-based sensors or biosensors, molecularly imprinted sensors, or other host-guest-like systems and non-enzymatic sensors [6,20].

The detection principle of immunoassays exploits strong immunogenic interaction between an antibody and its corresponding antigen. In the case of pesticide detection, pesticide molecules are usually bound to a larger molecule because, alone, they cannot provoke an immunogenic reaction due to their lower molecular weights. The analyte concentration is then correlated with the amount of labelled antigen or antibody to the corresponding ligand coated on the transducer surface [6]. Most pesticides are inhibitors of cholinesterases, enzymes, which are critical in Neurobiology, Toxicology, and Pharmacology. Among them, acetylcholinesterase (AChE), which is crucial for nerve impulse transmission in humans, is mostly reported to be used for the electrochemical detection of pesticides. Electrochemical biosensors thus exploit the interaction between the selected pesticide and the specific enzyme, which is usually immobilised onto different nanostructured materials [6,20,26,27,28,29,30]. Although enzyme-based sensors, in most cases, exhibit excellent detection performance for pesticides, there are still severe limitations regarding their fabrication, storage, stability, and their limited lifetime due to the denaturation of the biological material present on the electrode’s surface. In order to avoid the use of unstable enzymes and various natural antibodies, molecularly imprinted polymers (MIPs) are gaining much attention as an alternative for preparing molecular recognition systems. In general, first, MIPs are prepared via polymerisation of the selected monomer in the presence of a target molecule or template, i.e., the analyte of interest, e.g., pesticide. Then the target is removed chemically, and cavities with the exact size, shape, and corresponding functional groups that are capable of rebinding the target molecule are formed [6,7,31,32].

The above-mentioned electrochemical sensing techniques have attracted considerable attention due to their high sensitivity, low cost, and inherent miniaturisation. However, there are still limitations regarding the use of antibodies or enzymes due to their reduced chemical and/or physical stability, which prevent the use of those recognition elements in harsh environments (organic solvents, acidic or basic medium, high temperatures, etc.) and complicated challenges in the development of high-affinity antibodies that are specific to a particular pesticide [6]. Moreover, the use of specific chemical or biological recognition elements (antibody, enzyme) usually results in the detection of a single and specific pesticide, and, therefore, developing a sensor for simultaneous determination of multiple pesticides is strongly recommended.

In this regard, another group of electrochemical sensors that do not require any biological or synthetic recognition elements and still exhibit high sensitivity and selectivity, are cost-efficient and enable on-site analysis with high accuracy are introduced, i.e., non-enzymatic electrochemical sensors [19,33,34,35].

In the case of non-enzymatic electrochemical sensors, modifying the surface of the working electrode, where the electrochemical reaction takes place, becomes crucial. To improve the charge transfer between the electrode and the analyte and, thus, increase the sensing of the selected analytes, developing nanotechnology is gaining much attention. Considerable attention has been given to developing non-enzymatic electrochemical sensors modified by various functional nanomaterials. The introduction of nanomaterials in electrochemical sensors is a highly efficient analytical tool for detecting and quantifying pesticides. Therefore, nanoscale materials have been used in electrochemistry to modify the electrodes due to their large specific surface area, small size, uniform pore structure, and high loading capacity [36,37,38]. In addition to sensing, nanomaterials have also been employed to degrade and remove pesticides [39].

As presented in this work, nanocomposites promote the development of electrochemical sensors significantly. The simultaneous action between different components becomes essential and results in the superior properties of a nanocomposite, which are beyond the properties of each individual component. Following a recent paper by Lu et al. [37] on multivariate nanocomposites for electrochemical sensing in applications in food, nanocomposites can be divided into three main classes; binary, ternary, and multiple nanocomposites, depending on the number of components involved in the composite; such classifications will also be used in the present paper. The most common materials used in nanocomposites for modification of electrodes consist of metal (and/or its oxide) nanoparticles (NPs), carbon nanotubes or nanosheets, graphene-based materials such as graphene and (reduced) graphene oxide, and conductive polymers (most frequently Nafion and chitosan), as reported by Lu et al. [37].

**Figure 1:**
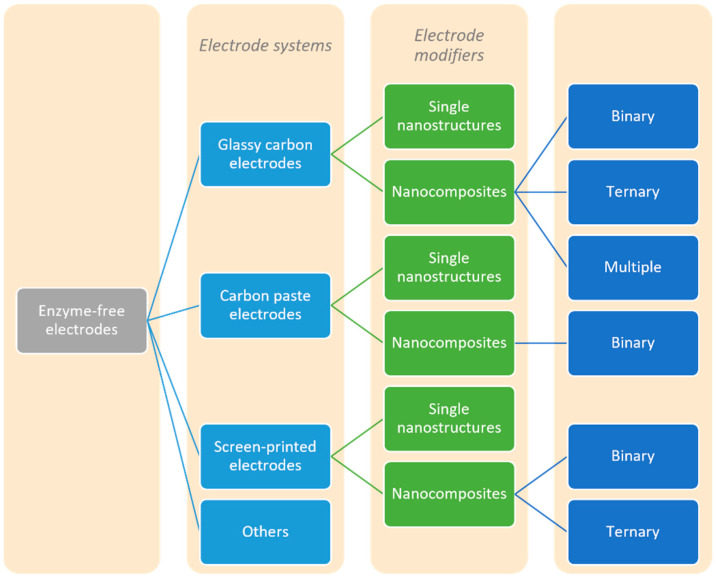
A schematic representation of the content reviewed in this work.

The existing studies and reviews primarily discuss the advances of biosensors, focusing on different types of electrode systems, lacking information about the non-enzymatic electrochemical sensors. Thus, the aim of this work is not only to give an overview of the specific area of non-enzymatic electrochemical sensing but rather to stress the improvement of the recently reported analytical methods for certain types of electrodes, i.e., glassy carbon electrodes, carbon paste electrodes, screen-printed electrodes, and some others, according to the type of the nanomaterial used as an electrode modifier, for all classes of pesticides. A comprehensive discussion is given for the studied electroanalytical methods, focusing on electrode modification and its analytical performance towards pesticide detection and quantification. A schematic representation of the electrodes and electrode modifiers represented in this work is shown in Figure 1.

## 2. The Use of Different Enzyme-Free Electrodes

This review focuses on the recently (since 2010) reported electrochemical systems for enzyme-free detection and quantification of pesticides using modified glassy carbon electrodes, carbon paste electrodes, screen-printed electrodes, and other types of electrodes, such as pencil graphite electrodes, gold electrodes, diamond electrodes and others. The emphasis is on the type of nanomaterial-based modifier of the electrodes, depending on the electrode system used for the electrochemical detection of pesticides.

The reported nanomaterials used as modifiers are either single NPs or a binary composite consisting of two different materials, based mainly on metal (oxide) and carbon. Next, the three- and multiple nanocomposites are discussed, including the role of other materials in electrochemical sensing, such as polymers, ionic liquids, carboxymethylcellulose, fullerenes and similar materials. All modifications are listed in the Tables, emphasising the analytical properties of the reported sensors. Special attention is given to the analytical performance of the different electrode-modified systems, such as linear concentration range, LOD, the limit of quantification (LOQ), sensitivity, selectivity, and applicability in the analysis of real samples. Finally, challenges and future perspectives are highlighted.

### 2.1. Glassy Carbon Electrode

Glassy carbon was first prepared in 1962 by Yamada and Sato [40] as a gas-impermeable carbon material with suitable properties, such as high chemical resistance, low thermal conductivity, high thermal expansion coefficient, and small pore size. Glassy carbon is generally prepared in various shapes by a controlled heating programme to temperatures above 1200 °C of a premodelled polymeric phenol-formaldehyde resin body in an inert atmosphere. It consists of smooth aromatic ribbon molecules which can stack above each other forming microfibrils that can twist, bend, and intertwine [41].

The application of glassy carbon material as an electrode in electrochemistry started with the discovery of the oxidation and reduction processes that occurred on the surface of glassy carbon electrodes (GCE) in aqueous solutions. Many researchers studied oxygen reduction processes, then the focus of the studies turned to the discovery of the differences in the electrochemical responses of pre-treated electrodes by polishing or by cathodic and anodic pre-treatments, and, later on, to analytical chemistry, i.e., to the study of the interactions of the GCE surface and selected analytes such as heavy metals, etc. [41,42].

Nowadays, GCE is used widely in electroanalysis due to its good electro(chemical) stability and high overvoltage for oxygen and hydrogen evolution reactions, performed by various techniques, such as voltammetry, stripping voltammetry, amperometry, potentiometry, and coulometry. Special attention is given to the oxidation and reduction processes of different organic and biological molecules in both aqueous and nonaqueous media.

Electrochemistry is based on interfacial interactions, and the modification of the GCE surface becomes crucial since it significantly affects the electrochemical parameters such as electron transfer rate, surface coverage, redox potential, etc. [43]. For this reason, the modifications of GCEs with various nanocomposites will be summarised within this section, and the influence of the electrode modification on the analytical performance of the sensors will be discussed.

#### 2.1.1. Modification of the Glassy Carbon Electrodes Using Single Nanostructures-Based Modifiers

The superior properties of single NPs that enable a large effective sensor surface area and fast electron transport make the modification of the electrochemical sensors essential for their use in electrochemical analysis [36,37]. However, only a few reports have been published since 2010 about the modification of GCEs with a single type of nanomaterials, as given in Table 1.

Carbon-based nanomaterials are very often used to modify the working electrodes in electrochemistry. Among them, carbon nanotubes (CNTs) present a promising candidate as an electrode-modifier for detecting pesticides due to their excellent stability in aqueous and non-aqueous solutions, good surface selectivity, fast charge transfer, and high mechanical strength [84]. For example, in the non-enzymatic electrochemical analysis of pesticides, Sundari et al. [44] showed that functionalised multi-wall CNTs (MWCNTs) enhanced the oxidation peak current of carbendazim (CBM) pesticide significantly as compared to bare GCE. A careful design of the MWCNTs-GCE-based sensor and optimised experimental conditions resulted in a very wide linear concentration range, i.e., 0.01–5·10^4^ µg L^−1^. The obtained LOD of 0.01 µg L^−1^ is the lowest LOD value reported for the modifications of the GCE by a single type of nanomaterial.

Metal-oxide nanostructures are another example of the GCE-modifier used for electrochemical detection of one of the well-known organophosphorus pesticides, methyl parathion (MP) [45]. The surface of the GCE was modified with Gd_2_O_3_ hollow nanospheres, which were characterised by high chemical durability, thermal stability, a large bandgap, and high dielectric constant. The hollow nanospheres had a spherical topography with a diameter of 200 nm. Cyclic voltammetry (CV) studies showed that this electrode had higher sensitivity for the reduction reaction of MP compared to bare GCE, which showed the importance of surface modification. Consequently, a wide linear concentration range of 0.05–100 µM and a low LOD of 0.03 µM were reported using differential pulse voltammetry (DPV).

**Figure 2:**
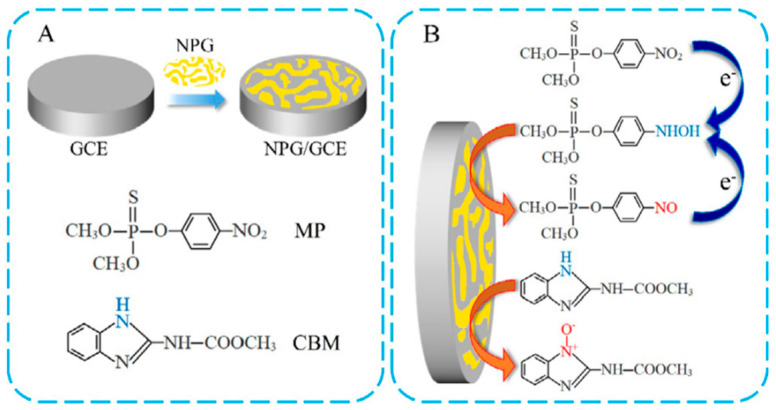
Schematic presentation of the (**A**) modified GCE by nanoporous gold (NPG) used for simultaneous detection of carbendazim (CBM) and methyl parathion (MP) pesticides, and (**B**) electrochemical reaction principles of both pesticides on the surface of the NPG-GCE. Reprinted with permission from Ref. [46]. Copyright 2019 Elsevier Ltd.

Very similar LOD values and linear concentration ranges were also reported when using nanoporous gold [46] as a modifier. The latter is studied regularly for electrochemical detection of oxygen, hydrogen peroxide, glucose, etc., due to its large specific surface area, high conductivity, strong adsorption, and high electrocatalytic activity [85]. Gao et al. [46] deposited a 100 nm-thick Au film with approximately 35 nm-large pores, and used it for simultaneous detection of two pesticides. This is the only study based on single NPs utilised as modifiers of GCE to determine two pesticides simultaneously. A schematic presentation of the electrode preparation and the proposed electrochemical reactions of both pesticides on the surface of the modified electrode is summarised in Figure 2. The authors found two well-separated current peaks for MP and CBM, i.e., +0.25 V vs. SCE for MP and +0.95 V vs. SCE for CBM, which enabled their simultaneous determination. The linear concentration ranges of 3–25 µM and 10–70 µM were obtained for MP and CBM, respectively. The reported LOD value for MP was 0.085 µM, while a higher LOD of 0.27 µM was reported for the CBM pesticide [46]. Since a simultaneous electrochemical determination of multiple pesticides is still scarce, the work on nanoporous gold as a modifier of GCE [46] shows the importance of metal NPs for further electrochemical investigations on enzyme-free sensors and their possible implementation into composites.

#### 2.1.2. Modification of the Glassy Carbon Electrodes Using Binary Nanocomposites-Based Modifiers

Even though some single nanomaterials have been reported as modifiers of the sensor’s surface, it is the most promising way to overcome the shortcomings of individual components, such as poor intrinsic conductivity and a tendency to agglomeration, to implement advanced nanomaterials into composites. Various types of nanocomposites were considered promising candidates for enzyme-free electrochemical detection of pesticides due to the synergistic effects of different (nano)materials, primarily carbon-based and metal(oxide)-based materials (Table 1). An overview of the modifications based on binary nanocomposites is presented below.

In the search for materials with a water-soluble nature, high electron transport activity, and high mechanical strength, graphene has been employed as a carrier material of metal, polymer and organic compounds to form a composite that would improve the stacking phenomenon of graphene and, consequently, the electrochemical performance of the modified sensors [37,84]. An electrochemical sensing ability for MP determination was studied recently using MoS_2_-graphene nanosheets (NS) [47]. Compared with the bare GCE, graphene/GCE, and MoS_2_/GCE, the use of MoS_2_/graphene NS nanocomposite improved the electroanalytic ability of the sensor significantly, especially its sensitivity. This phenomenon was ascribed to the primarily exposed electrochemically active area of the 3D network of the nanocomposite, high conductivity and excellent synergy between the graphene and MoS_2_ NS, i.e., through the possible π stacking interaction between the phenyl group of the MP pesticide and rich π electron density of the graphene. Also, the 3D graphene-CuO NP [48] and graphene-nickel-iron phosphosulfide (NiFeSP) [49] nanocomposites were used to improve the LOD and the sensitivity of the electrochemical detection of malathion and paraoxon ethyl pesticides, respectively.

**Figure 3:**
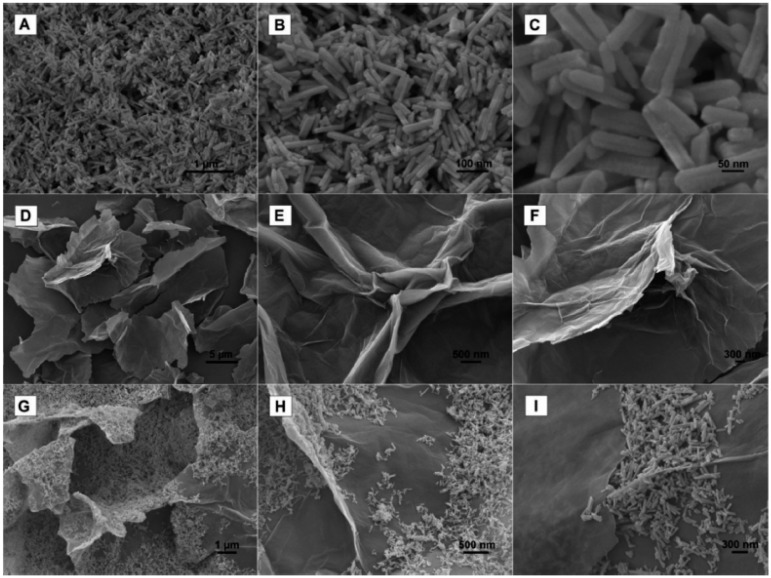
FE-SEM micrographs of the GdO nanorods (**A**–**C**), graphene aerogel (**D**–**F**) and nanocomposite consisting of GdO nanorods and graphene aerogel (**G**–**I**). Reprinted with permission from Ref. [86]. Copyright 2020 American Chemical Society.

In a recent paper, Suresh et al. [84] systematically reviewed the studies on the electrochemical detection of CBM pesticides by graphene-based hybrids. For example, GdO nanorods decorated on graphene aerogel were used as a modifier of the GCE [86]. The synthesized nanocomposite consisted of well-separated GdO nanorods with a diameter of approximately 50 nm and a length of below 400 nm, tightly anchored on the graphene aerogel matrix. The corresponding field-emission scanning electron microscopy (FE-SEM) measurements of the GdO nanorods, graphene aerogel, and the final composite are shown in Figure 3. The fabricated nanocomposite enhanced the electrochemical performance of the prepared sensor greatly, which had a low LOD (3 nM) and a wide linear concentration range of 0.01–75.00 µM, good selectivity, reproducibility, and storage stability [86]. In another study, N-methyl-2-pyrrolidone exfoliated graphene was used for CBM detection because of its increased electrode surface area, resulting in an increased number of reactive sites that led to higher accumulation of CBM and faster electron transfer [87]. This sensor had a narrower linear concentration range (0.00523–1.569 µM) as compared to the previous work on GdO-graphene-GCE [86] and a very low LOD of 0.78 nM. In addition, graphene oxide was proven to enhance the electrochemical performance of the sensors in combination with cyclodextrins [88], nanoporous copper [89], and MWCNTs [90], which was attributed to the synergetic effect of the unique properties of both components of the binary composite.

One of the lately most important carbon-based materials, CNTs, were prepared as binary composites with different components, such as metal-oxides [50,57,79], polymers [51,52], carboxymethyl cellulose (CMC) [54], MoS_2_ quantum dots [55], other carbon-based nanostructures [53,54], etc. For example, a fast and easy-to-follow method for fabrication of the modified GCE sensor was proposed by Ghodsi et al. [50], where the most sensitive electrode for detecting diazinon pesticide was obtained for the MWCNTs-TiO_2_ nanocomposite as compared to the bare GCE, MWCNTs/GCE, and TiO_2_/GCE electrodes. A low LOD of 3 nM and a linear concentration range of 11–8360 nM, were obtained using the MWCNTs-TiO_2_-modified GCE.

Three-dimensional MIP-coated CNTs were recently utilised for profenofos insecticide detection [51]. Functionalised CNTs with silica were characterised with a diameter of approximately 14.3 nm and uniform coverage of the MIP lamella throughout the CNT tubular structure. The as-prepared nanocomposite increased the surface area significantly, leading to an increased number of imprinting sites, resulting in improved sensitivity and electron transfer. The LOD and LOQ obtained by implementing 3D CNT-MIP nanocomposite in an amperometric sensor were 2 nM and 7 nM, respectively. Low LOD and LOQ values and a very wide linear concentration range, i.e., 0.01–200 µM, were ascribed to the characteristics of the carefully designed nanocomposite.

Immense analytical improvement in electrochemical sensing was achieved with the implementation of conductive polymers into the sensing devices. Here, the role of polymers is to provide sufficient electrochemical conductivity to transduce the occurrence of the coupling event into the analytical signal [91,92]. Due to the different properties of conductive polymers, such as their inherent redox activity, electronic and ionic conductivities, conformational and structural changes, polymers may be involved in electrochemical reactions, making them selective agents and transducers at the same time. The crucial role of a polymer in non-enzymatic electrochemical sensing of pesticides was also investigated by Prasad et al. [52], who found that only when the prepared single-walled CTNs (SWCNTs) were dispersed in a Nafion solution did the reduction peak current of the dicapthon pesticide significantly increase, as a result of the good aspect ratio and strong adsorption ability of the prepared modifier.

A composite consisting of two different types of carbon structures, namely, hydroxylated multiwall carbon nanotubes (HMWCNT) and single-wall carbon nanohorns (CNH), was recently prepared for electrochemical determination of nitenpyram, a nicotinamide insecticide [53]. CNHs are a new kind of carbon nanostructures that can be recognised by their irregular cylinders made of a single layer of graphene curled together that have a diameter of 2–5 nm. Similar to other carbon-based nanostructures, the main advantages of CNH for electrochemical sensing are their small particle size, heterogeneous surface and multiple reactive sites. A good electrochemical response of the HMWCNT-CNH-based GCE was reported, and a broad linear concentration range of 20–2000 nM with an LOD value of 4.0 nM. In another paper, CMC-functionalised MWCNTs were employed successfully in the electrochemical detection of CBM pesticide, owing to the enhanced adsorption capacity of CNTs toward the target pesticide via the abundant hydroxyl and carboxylic groups in the CMC [54]. This sensor showed a wider linear concentration range (0.03–10 µM) as compared to previous work on HMWCNT-CNH-based GCE [53], high sensitivity of 6.588 µA µM^−1^ and an LOD of 0.015 µM.

A combination of two metal-oxides was also reported as a modifier of GCE for the detection of malathion [59]. The large specific surface area of the porous CuO-CeO_2_ nanocomposite and good adsorption capacity of CuO particles with the P=S bond of malathion molecules resulted in a sensitivity of the CuO-CeO_2_-GCE sensor of 2.07 µA nM^−1^ cm^−2^ and a very low LOD of 3.3 fM. The reported linear concentration range was 10 fM–100 nM. The good analytical response was attributed to the fact that CuO and TiO_2_ can bind easily to organophosphorus pesticides with a high affinity to the phosphate groups in the pesticides. In another study, CuO nanowires (NW) combined with SWCNTs were also used for the detection of malathion [93].

Acetylene black is another carbon nanomaterial characterised by high electrocatalytic activity, high conductivity, and an accumulation capacity for many organisms. A nanocomposite based on the use of acetylene black-chitosan film and a polymer was reported to detect MP [58]. Polymer chitosan is another crucial material for electrochemical sensing due to its membrane-forming ability, biodegradability, biocompatibility, and high mechanical strength. In the case of acetylene black-chitosan nanocomposite, chitosan dispersed acetylene black successfully and prevented its agglomeration, providing more active sites for the electrochemical reaction of MP. Consequently, the modification of GCE resulted in the increased sensitivity of the proposed sensor as compared to the bare electrode. In addition, also the very low LOD of 2·10^−9^ M shows a vital manner to improve the analytical properties of electrochemical sensors based on GCE [58].

The applicability of magnetic NPs as a constituent of a binary nanocomposite was also proven for non-enzymatic electrochemical sensing. Miao et al. [64] reported the implementation of Fe_3_O_4_ NPs into a nanocomposite with polydopamine MIP, used in a sensor for sensitive determination of dichlorodiphenyltrichloroethane (4,4′-DDT) insecticide. Here, EIS was employed to investigate the modified electrodes’ interface properties and to evaluate the proposed sensor’s electrochemical response. Since the electrochemical impedance increased with the adsorption of the 4,4′-DDT, the relation was studied between both. The method showed a linear correlation between the charge transfer resistance (*R*_ct_) and the 4,4′-DDTconcentration in the range of 1·10^−11^–1·10^−3^ M, with a very low LOD of 6·10^−12^ M.

**Figure 4:**
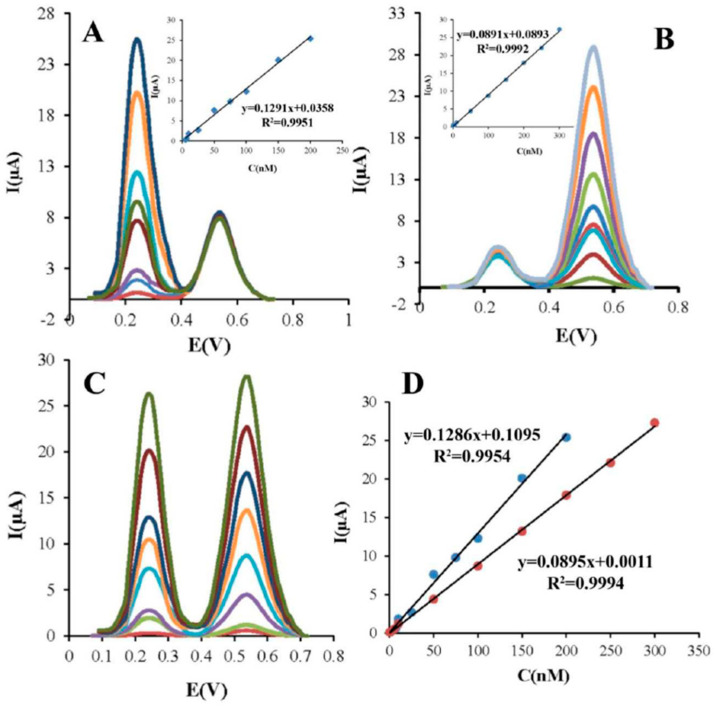
DPV results for (**A**) The solution containing an increasing concentration of carbofuran (CBF) (current peak at +0.23 V) in the range from 5.0 to 200.0 nM and a constant concentration of 100 nM of carbaryl (current peak at +0.51 V), (**B**) The solution with variable concentrations of carbaryl from 1.0 to 300 nM, and a constant concentration of CBF, (**C**) The solution containing a variable concentration of both pesticides and (**D**) The corresponding linear concentration ranges for both pesticides. All measurements were performed using MIL(Fe)101-rGO-modified GCE in the B-R buffer solution. Reprinted with permission from Ref. [66]. Copyright 2019 Wiley-VCH Verlag GmbH & Co. KGaA.

Only one study based on simultaneous detection of pesticides utilising a binary composite was performed based on metal-organic frameworks (MOFs), namely, iron-carboxylate nano MOFs MIL(Fe)-101 and MIL(Fe)-53 [66]. MOFs consist of metal nodes connected by organic ligands, with structures ranging from microporous to mesoporous, and can be used for electrochemical analysis due to their porous and flexible structure, large specific surface area, high catalytic activity, and thermal and chemical stability [94]. Soltani-Shahrivar et al. [66] combined MIL(Fe) with reduced graphene oxide (rGO) into new nanocomposites, i.e., MIL(Fe)53-rGO and MIL(Fe)-101-rGO, which resulted in increased electrical conductivity and sensitivity of the modified electrodes. Due to a more than two times higher current peak response of the MIL(Fe)-101-rGO-GCE than MIL(Fe)-53-Rgo-GCE, the first system was used to determine two pesticides simultaneously. The results are shown in Figure 4. The sensor exhibited two linear concentration ranges, i.e., 5.0–200.0 nM for CBF and 1.0–300.0 nM for CBR. The obtained LOD values of 0.52 and 0.11 nM for the CBF and CBR were comparable to the LOD values obtained when analysed individually, as given in Figure 4C,D, which showed the applicability of the modified GCE-based sensor for simultaneous determination of multiple pesticides.

#### 2.1.3. Modification of the Glassy Carbon Electrodes Using Ternary Nanocomposites-Based Modifiers

Ternary nanocomposites are very often used as modifiers of the GCE for the electrochemical detection of pesticides, as summarised in Table 1. It is evident that ternary nanocomposites consist mostly of a carbon-based material, either (reduced) graphene oxide, carbon nanotubes, nanowires, or nanosheets, and are usually used in combination with metal (oxide) NPs and a conductive polymer.

An example of a GO-based ternary nanocomposite is composed of ZnO NPs, GO NPs, and polymer polyaniline (PANI) [67]. A GCE covered with the ZnO-PANI-GO nanocomposite showed a 100-times higher current response of the oxidation peak of imidacloprid pesticide compared to the bare electrode. The LOD and LOQ values of 1.3·10^−8^ M and 1.3·10^−7^ M were reported, respectively, with a linear concentration range of 1.2·10^−7^–2.1·10^−6^ M. In another study, rGO was employed for detection of MP, where a nanocomposite consisting of MnO_2_, polythiophene (PTH) and rGO was prepared by the in situ chemical oxidative polymerisation method [68]. Here, the sensor had a wide linear concentration range of 10 nM–1 µM, with a low LOD of 5.72 nM.

An electrochemical co-deposition method was recently utilised to synthesise the Au-ZrO_2_-graphene nanosheets (GNS) nanocomposite for electroanalysis of MP [69]. When a ternary composite Au-ZrO_2_-GNS was synthesised thoroughly, Au NPs with an average diameter of 20 nm were distributed homogeneously on the ZrO_2_-GNS film, and no agglomeration of the particles was observed. Such morphology of the modifier resulted in an enhanced current response. The latter was ascribed to the large surface area provided by the graphene that enhanced the electron transfer, which was supported by the EIS analysis, where the lowest *R*_ct_ was determined for the Au-ZrO_2_-GNS composite. The current response of the reduction peak significantly increased when ZrO_2_ was present in the composite, providing evidence that the ZrO_2_ absorbed the molecules of the pesticide successfully, due to its high binding affinity towards the phosphorous group in MP. The authors found that the electron exchange can be hindered significantly between the pesticide and Au-ZrO_2_-GNS when a too thick layer of Nafion is deposited on the top of the nanocomposite. Finally, all these facts contributed to the enhanced electrochemical response of the systematically designed Au-ZrO_2_-GNS-based sensor: Low LOD of 1 ng mL^−1^ and linear concentration ranges of 1–100 ng mL^−1^ and 100–2400 ng mL^−1^ [69]. In another work, an Au NPs-chitosan-GNS nanocomposite was also designed for the detection of MP pesticide. Such modification facilitated the preconcentration of MP and thus enhanced the stripping current response of the analysed pesticide. The proposed sensor exhibited a slightly lower LOD value (0.6 ng mL^−1^) as compared to the previous work on the Au-ZrO_2_-GNS-based sensor [69], and narrower linear concentration ranges (0.001–0.1 µg mL^−1^ and 0.2–1.0 µg mL^−1^).

**Figure 5:**
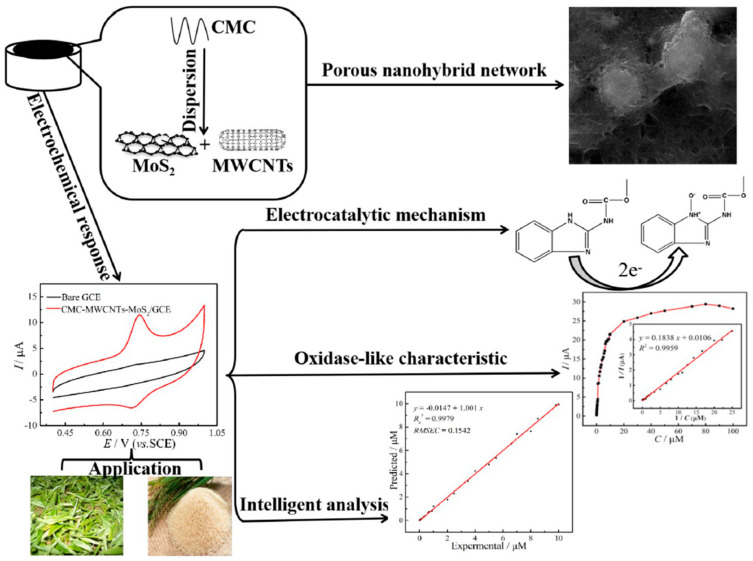
Schematic presentation of the electrochemical characterization of the CMC-MWCNTs-MoS_2_-GCE-based electrochemical sensor used to detect carbendazim pesticide in tea and rice samples. Reprinted with permission from Ref. [73]. Copyright 2020 Elsevier B.V.

In addition to GNS, CNTs proved to be promising carbon-based candidates for ternary nanocomposites. For example, the sensitivity of the Pd-MWCNTs-Nafion-GCE was three times higher than for MWCNTS-Nafion-GCE, which showed the excellent synergistic effect of Pd NPs and MWCNTs, and, thus, also the importance of metal NPs in electrochemical sensing [71]. Similarly, Canevari et al. [72] utilised MWCNT, SiO_2_ and RuPc in a nanocomposite to detect the fenitrothion insecticide. The current response of the MWCNT-SiO_2_-RuPc modifier was higher as compared to MWCNT-SiO_2_, which was ascribed to the increased active area of the modifier that promoted the electron transfer at the electrode–solution interface. Moreover, the study showed that the presence of RuPc in a composite slowed down the saturation of the electrode’s surface, as compared to the MWCNT-SiO_2_-modified GCE. The advantages of MWCNTs for electrochemical detection of pesticides were also exploited in a CMC-MWCNTs-MoS_2_ composite for CBM detection [73]. Here, electrochemically-active graphene-like MoS_2_ offered increased surface area and the possibility for simple surface modification, while the CMC exhibited water processability, synergistic electrocatalytic ability and enhanced adhesion and stability of the electrode. All these factors contributed complementarily to the enhancement of the electrochemical response of the CMC-MWCNTs-MoS_2_-based sensor with an LOD of 7.4 nM and a wide linear concentration range of 0.04–9 µM. Furthermore, the reciprocal of the CBM concentration was in proportion to the reciprocal of the peak currents at higher CBM concentrations, in the range of 10–100 µM. The results are schematically shown in Figure 5.

The possibility of acting as an electron mediator for activation of oxidations or reductions of the target substances makes fullerene and its derivates attractive for electrochemical analysis [95]. In work by Teadoum et al. [74], a fullerene-MWCNTs-Nafion nanocomposite was synthesised and used as a GCE-modifier for the detection of CBM. The studied sensor was characterised by low LOD and LOQ values, i.e., 1.7·10^−8^ M and 5.57·10^−8^ M, respectively. The linear response of the sensor towards CBM pesticide was narrow, in the low concentration range of 2·10^−8^–3.5·10^−7^ M; nonetheless, high sensitivity of 419.69 A M^−1^ was reported.

The electrochemical properties of MWCNTs were utilised in a design of an IL-CaF_2_O_4_-MWCNTs-based composite for the detection of CBM pesticide [75]. The 1-propargyl-3-butyl imidazolium bromide IL was immobilised on the surface of CaF_2_O_4_ NPs, to protect the agglomeration of NPs and improve the compatibility and dispersibility of the prepared composite when designed with MWCNTs. The carefully designed composite enhanced the charge-transfer-activity of the proposed sensor greatly. According to the EIS analysis, *R*_ct_ decreased significantly, from 530 Ω to 180 Ω, as the ILs-CaFe_2_O_4_ was introduced as a modifier of the GCE. Moreover, *R*_ct_ decreased further to 105 Ω when MWCNTs were incorporated into the composite, facilitating electron transfer between the electrode and the modified film. The corresponding sensor showed a linear response in two concentration ranges, i.e., 3.14·10^−8^–1.05·10^−5^ M and 1.05·10^−5^–1.05·10^−4^ M, with higher sensitivity in the low concentration range and a low LOD of 9.41 nM.

**Figure 6:**
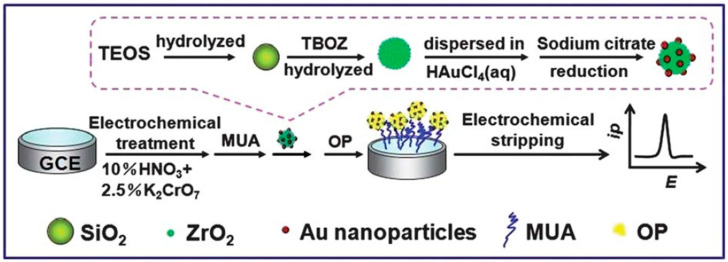
Schematic presentation of the development of the electrochemical sensor for detection of paraoxon-ethyl pesticide, which proceeds via paraoxon-ethyl adsorption and electrochemical stripping detection of the adsorbed pesticide. The scheme includes individual synthesis steps to prepare the Au-ZrO_2_-SiO_2_ nanocomposite, used as a modifier of the GCE. TEOS—tetraethoxysilane, TBOZ—zirconium n-butoxide, MUA—11-mercaptoundecanoic acid, OP—organophosphorous pesticides. Reprinted with permission from Ref. [76]. Copyright 2012 The Royal Society of Chemistry.

Ternary-based nanocomposites composed of two metal oxides in combination with Au NPs [76] and a polymer [77] were also prepared for electrochemical detection of pesticides. As an example, the synthesis of Au-ZrO_2_-SiO_2_ spheres [76] proceeded via the preparation of SiO_2_ spheres and ZrO_2_ NPs, separately. In the next step, the ZrO_2_ NPs were dispersed carefully on the surface of the SiO_2_ spheres, forming a very thin shell layer. Finally, Au NPs were deposited on the surface of the ZrO_2_-SiO_2_ spheres, as presented schematically in Figure 6. The final Au-ZrO_2_-SiO_2_ nanocomposite consisted of the Au NPs with a diameter of 20 nm, distributed uniformly on the surface of the ZrO_2_-SiO_2_ spheres. The electrochemical detection of paraoxon-ethyl occurred in two main steps: Firstly, the pesticide was adsorbed on the electrode surface, and then the current was measured in the stripping step. The current response increased with an increasing paraoxon-ethyl concentration in the range of 1.0–500 ng mL^−1^, with an LOD of 0.5 ng mL^−1^. Moreover, no interferences of other electroactive nitrophenyl derivatives, such as nitrobenzene, nitrophenol, and oxygen-containing inorganic ions, were observed in the proposed sensor.

In addition to metal oxides such as ZrO_2_, SiO_2_, CuO, and TiO_2_, magnetic Fe_3_O_4_ NPs were used in a ternary nanocomposite to detect methomyl, a member of the carbamate pesticides [78]. More importantly, the magnetic composite ZnFe_2_O_4_-SWCNTs-Nafion was synthesised and implemented into an electrochemical sensor to detect CBM and thiabendazole (TBZ) pesticides simultaneously. The ZnFe_2_O_4_-SWCNTs composite consisted of 30 nm-large ZnFe_2_O_4_ NPs that were dispersed uniformly on the surface of the SWCNTs. The EIS study confirmed that the electron exchange was enhanced greatly when the composite was used as a modifier. Consequently, the ZnFe_2_O_4_-SWCNTs-GCE sensor exhibited excellent electrochemical performance with a wide linear concentration range (1.0–100.0 µM) for both pesticides and low LOD values (Table 1). Here, a surfactant cetyltrimethylammonium bromide was used, which contributed to the enhanced electrochemical signal via electrostatic interaction that increased the absorption of CMB and TBZ on the surface of the electrode.

The importance of ternary composites for electrochemical detection of pesticides was also proven by the use of a CoO-decorated rGO nanocomposite for simultaneous determination of two carbamate pesticides, CBF and CBR [80]. The GCE was covered with CoO NPs with a sphere-like morphology, that appeared homogeneously on the surface of the rGO sheets dispersed in a Nafion solution. The sensor had wide linear concentration ranges of 0.2–70 µM and 0.5–200 µM for CBF and CBR pesticides, respectively. The LOD values were 4.2 μg/L (1.90·10^−2^ µM) for CBF and 7.5 μg/L (3.73·10^−2^ µM) for CBR, respectively, as reported by Karimian et al. [80].

In another study, Karimian et al. [81] modified GCE with a nanocomposite consisting of MOF UiO-66, TiO_2,_ and graphene oxide (shortly UiO-66-TiO_2_-GO) and applied it to the simultaneous detection of two organophosphorus pesticides, paraoxon and chlorpyrifos. Compared with the bare GCE, the UiO-66-TiO_2_-GO nanocomposite exhibited faster electron transfer on the electrode’s surface due to the large effective surface area of the composite. This method showed a linear response in narrower concentration ranges compared to the previous work based on CoO-decorated rGO nanocomposite [80]; concentration ranges of 1.0–100.0 nM and 5.0–300.0 nM were obtained for the paraoxon and chlorpyrifos pesticides, respectively. The reported LOD values were 0.22 nM for paraoxon and 1.20 nM for chlorpyrifos. The latter three case studies show crucial progress in the simultaneous determination of two pesticides, which was achieved by using carbon-based ternary nanocomposites as modifiers.

#### 2.1.4. Modification of the Glassy Carbon Electrodes Using Multiple Nanocomposites-Based Modifiers

Two studies were found in the case of multiple nanocomposites that reported their use as modifiers of the GCEs for the detection of pesticides. In the first case, Tan et al. [82] reported the MIP-based composite prepared in combination with rGO and Au NPs to detect the CBF pesticide. First, the rGO-Au nanocomposite was synthesised, dispersed in a Nafion-ethanol solution, and then dropcasted onto the GCE’s surface. In the next step, the MIPs were prepared, and the mixture was dropcasted onto the rGO-Au-GCE surface and polymerised. The CBF template was removed from the system by washing with an acetic acid/ethanol solution in the last step. The prepared MIP-rGO-Au-GCE exhibited a linear concentration range of 5·10^−8^–2·10^−5^ M, with an LOD of 0.02·10^−6^ M. The sensor exhibited very high selectivity towards CBF, which is ascribed mainly to the imprinted effect used in the fabrication of the sensor.

In another study, a nanocomposite consisting of MWCNTs, TiO_2_ NPs, and carboxymethyl chitosan (CMCh) moieties was used as a modifier for the detection of trichlorfon, another member of the organophosphorus pesticides [83]. The use of the MWCNTs-TiO_2_-CMCh modifier resulted in a slightly higher LOD value (0.40 µM) as compared to the previously reported work by Tan et al. [82], where the LOD of 0.02 µM was reported for the MIP-rGO-Au-GCE. Nonetheless, compared to MIP-rGO-Au-GCE [82], the use of MWCNTs-TiO_2_-CMCh resulted in a wider concentration range and higher sensitivity of the sensor.

### 2.2. Carbon Paste Electrode

Carbon paste, defined as a mixture of carbon (graphite) powder and a binder, is a very widely used electrode material used for various applications, such as electrodes, sensors, and detectors. A suitable carbon material, used as a carbon paste electrode, or, shortly, CPE, should possess the following properties: The particle size of the material should be in the lower micrometre range (around 5–20 µm), or, more recently, in nanometres, the distribution of particles should be uniform, the material should have high chemical purity and low adsorption capabilities. In addition, the binder, as the second compound in CPEs, contributes importantly to the functional properties of CPE. The chemical inertness, electrochemical inactivity, high viscosity, low volatility, insolubility in aqueous solution, and immiscibility with organic solvents are the required parameters of a suitable binder compound. The most commonly used binders in commercial CPEs are mineral (paraffin) oils and, in rarer cases, aliphatic and aromatic hydrocarbons, their derivatives, silicone oils are employed, and, more recently, room temperature ionic liquids [96].

The main advantages of CPEs are their simple construction and variability in size and shape. The original design by Adams [97] was based on a short Teflon rod with an opening at the end of the rod, where the paste was inserted into the housing with a Pt-wire inside the rod as a contact. Following the original design, simple structures of CPE-based sensors are, nowadays, used widely in practice, i.e., a micropipette tip filled with carbon paste, which contains a wire-contact, piston-driven electrode holders for the carbon paste, various other polytetrafluoroethylene (PTFE) tubes, glass tubes, syringe needles, etc., where the end-holes can be refilled easily with a new portion of the paste [96].

Similarly, as in the case of GCEs, modification of CPEs leads to the improvement of their electrochemical and analytical performance, as discussed below. Modifications of CPEs proceed in a simple way, either by in situ modification, by mechanical admixing of modifiers into the carbon paste bulk in solid-state, by dissolution in a pasting liquid, or by impregnation of carbon powder in the case when graphite particles are soaked with a solution containing a dissolved modifier. Carbon powder pre-treated in this way is, after evaporation of the solvent, mixed and homogenised with the selected liquid binder. Furthermore, the chemical pre-treatment of carbon powder or anodisation/cathodisation are certain manners of modification used in the case of CPEs [96,98,99].

Other advantages of CPEs, such as high stability, sensitivity, and selectivity, small size, and low cost, make CPEs useful in a variety of research areas, including the electrochemical analysis of various inorganic and organic ions, complexes, and molecules. Bare/unmodified CPEs were studied for the non-enzymatic detection of different pesticides, such as glyphosate [100], fenhexamid [101], CBM [102], and linuron [103], as summarised in Table 2. For example, Brycht et al. [101] prepared a CPE sensor for the determination of fenhexamid residues, which had a linear concentration range of 3.22–44.60 µM, with an LOD of 0.97 µM. In work by Oliveira et al. [100], the CPE sensor exhibited a broader linear concentration range as compared to the previous study [100], a lower LOD value, as well as greatly enhanced sensitivity (Table 2). A low LOD (0.96 µg L^−1^) was also obtained for the determination of CBM [102], and a wide linear concentration range of 25.75–309.02 µg L^−1^ was reported for the CPE for the determination of the linuron pesticide [103]. These studies have confirmed the applicability of unmodified CPEs for the non-enzymatic determination of pesticides. Furthermore, the electrochemical performance of the CPE-based sensor can be further enhanced by the introduction of nanomaterials as modifiers of the electrodes, as discussed in the next section.

#### 2.2.1. Modification of the Carbon Paste Electrodes Using Single Nanostructures-Based Modifiers

Single nanomaterials are regularly used modifiers of carbon pastes for non-enzymatic electrochemical sensing of pesticides, as summarised in Table 2. This is in contrast to the observed GCEs in the previous section, where binary and ternary nanocomposites were studied predominantly. For example, carbon paste prepared from graphite powder and paraffin oil was recently modified with MWCNTs and used for diazinon detection [104]. In addition to the irregular plates of graphite powder, the tubular shape of MWCNTs was confirmed on the surface of the modified CPE. The sensor showed a wide linear concentration range with low LOD (4.5·10^−10^ M), which is among the lowest values obtained for CPEs (see Table 2). In another study, the CPE-based sensor was prepared by mixing MWCNT powder with mineral oil for the determination of cyromazine, a triazine pesticide [105]. As opposed to the previous study [104], no graphite powder was used in this study. The authors found that the method exhibited a wider linear concentration range and higher sensitivity as compared to bare GCE, which confirmed the importance of CPE modification.

The influence of the electrode material on the analytical response of the sensors was recently studied for different carbon-based electrodes, such as GCE, GCPE, CPE, MWCNT-CPE, and SPCE [101]. Among the CPEs prepared for the determination of fenhexamid pesticide, the highest sensitivity was obtained for the bare CPE, while a much wider linear concentration range with lower LOD was obtained for the MWCNTs-CPE (Table 2) [101]. This study is another work among many [100,102,103,104,105,106,107,108,109,110,111,112,113,114,115,116,117,118,119,120,121,122] that proved the complexity of designing a CPE-based sensor with the desired analytical properties, such as low LOD, a wide linear concentration range, high sensitivity, and excellent selectivity, stability and reproducibility.

In addition to carbon-based nanostructures, metal-oxide NPs such as TiO_2_ and ZrO_2_ were implemented as modifiers into CPE-based sensors. Anatase TiO_2_-modified CPE was recently suggested as an alternative electrode for electrochemical detection of cypermethrin [106]. The TiO_2_-CPE nanocomposite exhibited a homogeneous microstructure with well-defined TiO_2_ NPs incorporated in the CPE matrix and decreased porosity as compared to bare CPE. Such modification of CPE resulted in a linear concentration range of 0.1–1 ppm with an LOD of 0.0978 ppm. Another study, where metal oxide ZrO_2_ NPs were employed in CPEs, was performed to detect the widely used pesticide MP [107]. The well-defined and high current peaks of the modified electrode were ascribed to the strong affinity of CPE toward the phosphate group of MP molecules, which resulted in a linear concentration range of 5–3000 ng mL^−1^, and LOD and LOQ values of 2 ng mL^−1^ and 5 ng mL^−1^, respectively. In addition, Fe_3_O_4_ NPs showed their potential as modifiers of CPEs in non-enzymatic electrochemical sensing of the chlorpyrifos pesticide [108].

The elemental doping of nanomaterials is another innovative approach to improving the electrochemical response of the CPE-based sensors [109,110]. It was found that heteroatom-doping of Nd_2_O_3_ caused defects in the crystal lattice that facilitated the electron transport and thus promoted the reaction between the modified electrode and the target molecule [110]. The resulting sensor had a wide linear concentration range of 0.08–50 µM and an LOD of 0.027 µM. An even lower LOD value and higher sensitivity were obtained for the sensor based on Ce-doped ZnWO_4_, with a narrow linear concentration range [109]. The controllable introduction of crystal defects, i.e., oxygen vacancies, and their role in the electrochemical oxidation process of CBM, and the resulting analytical performance are discussed in detail by Zhou et al. [109].

#### 2.2.2. Modification of the Carbon Paste Electrodes Using Binary Nanocomposites-Based Modifiers

Following the same trend as observed for single NPs-based modifiers, MWCNTs also have a crucial role in the electrochemical performance of the CPEs when modified with binary nanocomposites. For example, Table 2 shows that a very wide linear concentration range was achieved when MWCNTs were combined with MIP for the detection of different pesticides, such as diazinon [112], dicloran [113], and diuron [114], as recently discussed by Mostafiz et al. [123]. In general, the use of MIP as a modifier enables a sensor to have highly specific binding affinity towards the target compound(s) that results in high accuracy, selectivity, great reusability, and robustness of the sensors.

Very recently, other examples of metal oxide-MWCNTs-based composites, i.e., MWCNTs-ZnO [108] and MWCNTs-Ca-ZnO [109], were proposed to detect linuron and CBM pesticides, respectively. These compositions employ the advantages of high electrical conductivity, high surface area, and mechanical strength of MWCNTs, good selectivity, chemical stabilisation, and the good electron interaction characteristics of ZnO NPs. These facts led to the evolution of electrochemical sensors with high sensitivity, a wide linear concentration range and low LOD values, as seen in Table 2.

Iron(III) phthalocyanine (FePc) was firstly reported as an electrode modifier component in the electroanalysis of pesticides [117]. The FePc-MWCNTs-CPE sensor exhibited a higher anodic peak current than the MWCNT-CPE, MWCNTs-GCE, and even FePc-MWCNTs-GCE sensors [117], ascribed to the catalytic properties of the FePc. Correspondingly, the FePc-MWCNTs-CPE showed a good electrochemical response with high sensitivity and low LOD and LOQ values.

Metal oxides, such as FeO, TiO_2,_ and CuO, are another group of constituents of binary composites for non-enzymatic detection of pesticides, as reported by Nurdin et al. [118,119] and Amra et al. [120]. In Table 2, it is evident that metal oxides enable the development of the modified sensors with one of the lowest LOD values reported among the binary nanocomposite-based CPE sensors.

In a recent work by Özcan et al. [121], modification of the electrode proceeded via the preparation of Ag NPs and fumed silica (FS), which is a form of silicon oxide that is characterised by a high specific surface area in the amorphous form [121]. Here, the use of FS increased the adsorption of CBM to the electrode’s surface via molecular interactions such as dipole-dipole interaction and/or hydrogen bonding with surface silanol (-SiOH) groups. Moreover, the presence of Ag NPs in the nanocomposite contributed to the accelerated electron transfer during the electrochemical oxidation of CBM. These facts contributed complementarily to the enhanced electrochemical performance of the sensor, with a very low LOD value of 9.4·10^−10^ M and a wide linear concentration range.

**Figure 7:**
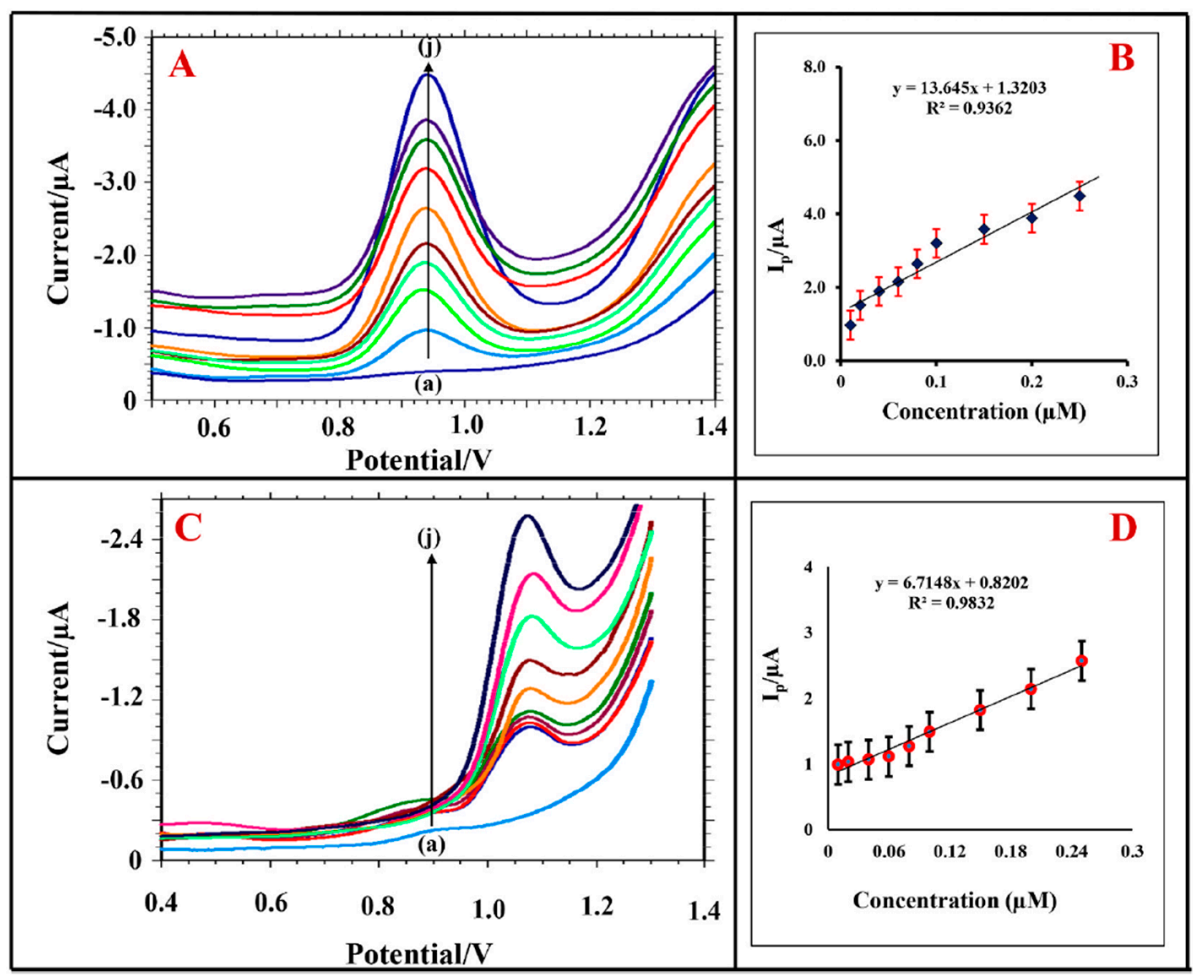
SW-voltammograms measured using g-C_3_N_4_-CTAB-modified CPE of (**A**) Amino-triazole and (**C**) Linuron herbicides. In (**B**,**D**), corresponding linear concentration ranges for both analytes are shown. Reprinted with permission from Ref. [122]. Copyright 2021 Elsevier Inc.

Another example, graphitic carbon nitride (g-C_3_N_4_) with cetyltrimethylammonium bromide (CTAB) surfactant, was introduced just recently as a CPE-modifier for the detection of two herbicides, linuron and amino-triazole [122]. The unique structure of the g-C_3_N_4_ nanosheets that provide more active sides and enhanced binding of the graphene-like structure (via a 2-D π layer structure with functional groups such as –NH–, =N– and –NH_2_) of g-C_3_N_4_ to both pesticides, are responsible for the enhanced electrochemical performance of the sensor. The corresponding voltammograms and linear concentration ranges are shown in Figure 7. The sensor showed very low LOD values, a very wide linear concentration range and a very high sensitivity for both pesticides.

### 2.3. Screen-Printed Electrode

Screen-printed electrodes (SPEs) are devices produced by printing ink on various substrates, mostly plastic or ceramics. Different inks are used to prepare all three electrodes, i.e., working, reference, and counter electrodes, on the same sensor. SPEs are prepared by the screen-printing technology, where a mesh screen with the defined structure is used, i.e., the size and shape of the electrodes. Viscous ink is then printed through the defined pattern on the mesh screen on the substrate and solidified during thermal treatment. In practice, several meshes are used to print different parts of the electrodes. In the last step, a shielding ink coating is used to insulate the conductive path of the electrodes [124,125].

Driven by the need for miniaturisation of analytical instruments, SPEs have been employed efficiently for rapid on-site analysis, which was possible due to the portability and simplicity of the SPE sensors and their excellent working performance. The ink used during the preparation of the SPEs, especially the working electrode, plays a significant role in the analytical performance of SPE sensors since both the selectivity and sensitivity of the method depend strongly on the properties of the ink used for printing the electrodes. Moreover, a simple modification of the electrodes makes SPEs of commercial importance and extremely attractive for various applications. The modification is possible either by changing the composition of the inks by adding different substances such as metals, polymers, complexing agents, etc., or by deposition of those substances on the surface of the manufactured electrodes [125,126].

In a review paper, Domínguez Renedo et al. [125] described the development of SPEs according to the type of materials used to modify the working electrode. Mostly, the unmodified screen-printed carbon-based electrodes (SPCEs), metal-based SPEs, film-coated, and enzyme-modified SPEs have been discussed for various applications in environmental analysis. In the case of film-coated SPCEs, mostly metal films such as Hg, Bi, Au, or Ni-based were used, in addition to some other materials, such as metallic NPs, cobalt phthalocyanine, nickel hexacyanoferrate, MnO_2_, etc. However, as will be seen throughout this section, various nanostructures and nanocomposites should also be considered when designing the SPEs, to improve their analytical response and their applicability for pesticide analysis.

#### 2.3.1. Modification of the Screen-Printed Electrodes Using Single Nanostructures-Based Modifiers

The most common approach for the modification of SPEs is based on various carbon nanomaterials and metal-oxide NPs, as seen in Table 3. Carbon black is another important nanomaterial that showed possible applications in various enzymatic biosensors and sensors, immunosensors, etc., of different analytes. The properties, such as excellent electrical conductivity, dispersibility in different solvents, the possibility of facile functionalisation, fast electron transfer kinetics and cost-effectiveness, make carbon nano black a suitable candidate as a modifier of SPE-based non-enzymatic sensors. The advantages of nano carbon black were exploited to fabricate an SPE sensor for the determination of four carbamate pesticides, i.e., carbaryl, isoprocarb, fenobucarb, and CBF [127]. The improved electrochemical sensing performance of the modified SPE compared to bare SPE was confirmed for all four analytes by means of a higher peak current and lower *R*_ct_. The modified sensor had a linear concentration range for all four pesticides analysed individually, in the range of 1.0·10^−7^–1.0·10^−4^ M with LOD values ≤ 8.00·10^−2^ µM. The authors showed the possible application of the modified sensor for the simultaneous analysis of pesticides of the two different molecular classes, i.e., carbaryl/carbofuran and isoprocarb/fenobucarb.

Similarly, as reported for GCE- and SPE-based sensors, metal- and metal oxide-based nanostructures were utilised in SPE sensors for non-enzymatic determination of pesticides. A well-defined and uniformly distributed hexagonal platelet-like structure of NiO NPs was observed on the surface of the NiO NPs-modified SPE determination of ethyl parathion [128]. The NiO NPs had an average size of NPs of 250 nm and exhibited a large specific surface area (S_BET_) of 150.1 m^2^ g^−1^ with bimodal meso-/macro-porous structures measuring 3.6 nm and 270 nm, respectively. The large specific surface area with a porous structure promoted electrocatalytic capability by ensuring the contact between the nanocrystal surfaces and the electrolyte molecules. The NiO NPs-modified SPE sensor exhibited linear response in two concentration ranges, i.e., 0.1–5 µM and 5–30 µM, respectively, with an LOD of 24 nM. Very similar LOD values were reported for the carbon-based modified SPE sensor, i.e., MWCNTs-based SPE for detection of CBM pesticide [129]. However, here, the method showed a linear concentration range of 4.00·10^−8^–4.01·10^−7^ M. The lowest LOD value (0.50·10^−3^ µM) reported among the single nanomaterials modifiers (Table 3) was obtained when the GO nanoribbons were utilised in the fabrication of the SPE [130]. The utilisation of GO nanoribbons resulted in a very wide overall linear concentration range of the sensor, i.e., 0.1–100 µM and 100–2500 µM.

The only study performed on SPE for simultaneous detection of pesticides was performed by Noyrod et al. [133]. Graphene-based SPE was designed to determine two pesticides, isoproturon and CBM. Two well-separated peaks were obtained, with linear concentration ranges of 0.02–10.0 mg L^−1^ for isoproturon and 0.50–10.0 mg L^−1^ for CBM, respectively.

#### 2.3.2. Modification of the Screen-Printed Electrodes Using Binary Nanocomposites-Based Modifiers

**Figure 8:**
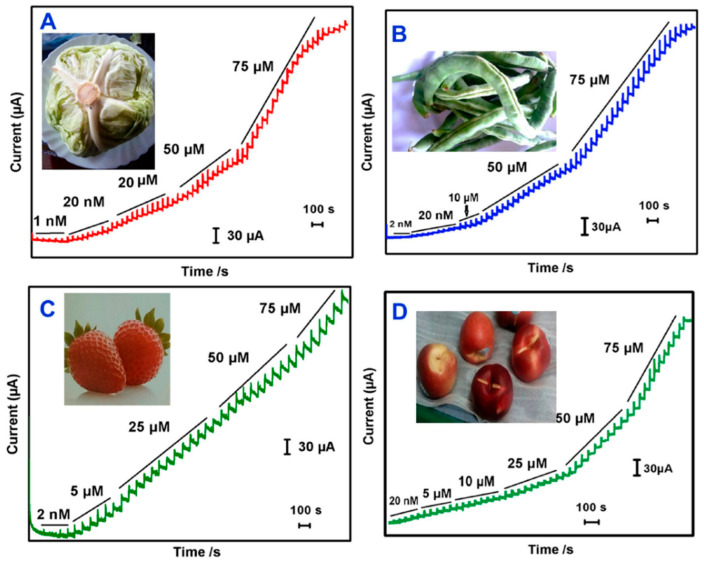
Amperometric responses obtained in the analysis of (**A**) Cabbage, (**B**) Green beans, (**C**) Strawberry, and (**D**) Nectarine real samples containing MP pesticide. The measurements were using Ag NPs-graphene nanoribbons-modified SPCE. Reprinted with permission from Ref. [134].

Following the same trend as observed for the GCE- and CPE-based sensor in Section 2.1 and Section 2.2, many researchers have reported on the modification of the SPEs with binary nanocomposites consisting of carbon-based materials as one or both constituents of the composite. For example, an electrochemical sensor based on SPCE modified with a nanocomposite consisting of Ag NPs supported graphene nanoribbons was developed to detect the MP pesticide [134]. The amperometric sensor had a very wide linear concentration range, i.e., 0.005–2780 µM and a very low LOD of 5.00·10^−4^ µM, which is the lowest value reported for the binary composites used as modifiers of SPEs (Table 3). The modified sensor was also applied to the determination of MP in four real samples, as shown in Figure 8. The excellent sensing performance of the sensor is ascribed to the synergic combination of both graphene nanoribbons and Ag NPs.

Another study on the carbon-based modified sensor was recently reported for the SCPE with three-dimensional zinc oxide NPs anchored on graphene oxide nanosheets (shortly ZnO-GO) [135]. ZnO NPs with an average size of 625 ± 10 nm had a crystalline star-like morphology and were interconnected on the surface of the GO nanosheets, well separated from each other. EIS analysis revealed that incorporating ZnO nanostars into the GO nanosheets resulted in an improved electron charge transfer performance of the electrode. Consequently, the modified sensor had a high sensitivity of 16.5237 μA μM^−1^ cm^−2^ and a wide linear concentration range of 0.03–669.65 μM, with LOD and LOQ of 1.12 nM and 8.61 nM, respectively. The GO nanosheets were also utilised with bimetal sulfide NPs, CuFeS_2,_ for the electrochemical detection of methyl paraoxon, primarily due to the high electrical conductivity of the nanocomposite [136]. The as-modified SPCE showed a similar linear concentration range (0.073–801.5 µM) and sensitivity (17.97 µA µM^−1^ cm^−2^) as compared to the previously described work on ZnO-GO-SPCE [135], with a slightly lower LOD value of 4.5 nm (see Table 3).

The metal carbide-based nanocomposite, NbC-Mo, was used as a binary modifier of the SPE for detecting the fenitrothion pesticide due to its largely exposed electrochemically active area of the 3D network, an excellent synergy between both materials and due to the high conductivity of the composite [137]. All these factors were complementary to the applicability of the NbC-Mo-SPE sensor, which had a very wide linear concentration range of 0.01–1889 µM, exhibiting a very low LOD value of 0.15 nM, but also low sensitivity of 0.355 µA µM^−1^ cm^−2^ among the reported SPE sensors prepared by binary composites.

The hydrophobic nature of pristine graphene and its tendency for self-agglomeration and restacking via Van der Waals interaction hinders the electrochemical response of the graphene-modified sensors significantly. To overcome these problems, the use of electrochemically-reduced micellar graphene oxide (ERMGO) was recently proposed as a modifier, with the cetyltrimethylammonium bromide (CTAB) as the surfactant [138]. By comparing different electrodes, i.e., unmodified SPE, ERGO, and ERMGO, the lowest *R*_ct_ was obtained for the ERMGO-modified electrode, implying the fact that CTAB orientation on the ERMGO’s surface facilitated the electron transfer process between the ERMGO’s surface and the electrolyte solution. These phenomena led to the improved electrochemical response of the SPE-based sensor for the detection of two pesticides, CBF and CBM, respectively. Moreover, such composition was applicable in the simultaneous determination of both pesticides. The ERMGO-based sensor had linear concentration ranges of 40–20,000 µg L^−1^ for CBF and 25–5000 µg L^−1^ for CBM, respectively.

#### 2.3.3. Modification of the Screen-Printed Electrodes Using Ternary Nanocomposites-Based Modifiers

In the case of the ternary nanocomposites, chitosan-functionalised carbon nanofibres supported Cu NPs (shortly Chitosan-fC-Cu) were used as a composite-modifier to determine the CBM pesticide [139]. The resulting sensor had a very wide linear concentration range of 0.8–277 µM and an LOD of 0.028 µM, ascribed to the high electrochemically active surface area of the composite and excellent electrical conductivity and good electrochemical stability. Interestingly, the composition consisting of rGO, NiS_2_, and curcumin NPs showed its applicability in the electrochemical sensing of pesticides [140]. The implementation of such a composition into the SPCE sensor was achieved via the already mentioned beneficial properties of rGO and excellent electrical conductivity of NiS_2_, and high electrocatalytic activity of curcumin NP, and, nevertheless, because of its facile preparation. The modification of the SPCE sensor for detection of the MP pesticide resulted in the linear concentration ranges of 0.25–5 µM and 5–80 µM, with an LOD of 8.7 nM. Moreover, the sensor showed applicability in the simultaneous detection of MP and another pollutant, 4-nitrophenol, used widely in industrial and agricultural applications.

### 2.4. Other Electrodes

Since 2010, various electrochemical sensors have been developed and applied successfully to different fields, including the sensing of pesticides. Substantial effort has been made to develop portable electrochemical sensors to determine the presence of pesticides in various real samples. When designing the electrochemical non-enzymatic sensors, mostly well-studied carbon-based electrodes such as GCE, SPCE, and CPEs were investigated, as was discussed in previous sections. However, due to the requirements for on-site detection, flexibility, fast analysis, and cost-effectiveness, new kinds of electrodes have been gaining much attention in recent years. In this regard, (un)modified graphite pencil electrodes, gold-based electrodes, diamond electrodes and others are discussed briefly in this section as examples, emphasising the material of the modifier and the analytical performance of the studied sensors.

Graphite pencils used for writing purposes were recognised as renewable, inexpensive, and readily available electrodes in late 1997. Nowadays, pencil graphite leads used as working electrodes are known as pencil graphite electrodes (PGEs). The electrode material is constituted by the commercially available graphite pencil leads with different hardnesses and blackness, denoted as 2B, 4B, HB, etc. The graphite leads are, in general, prepared by mixing natural graphite (75–80%) with an organic binder (13%) and spindle oil (8%) [141].

Similarities in the surface structure and kinetic behaviour between the PGEs and commercial carbon- and graphite-based electrodes were found in a systematic study by Kariuki [142]. For example, an HB pencil electrode exhibited comparable electron-transfer rates to those performed on a GCE electrode. Compared to other working electrodes such as GCEs, as an example, PGEs present lower background current contribution, higher sensitivity, good reproducibility, and simple modification of the electroactive surface area, permitting the analysis of low concentrations and small sample volumes without any deposition/preconcentration step [143]. PGEs have been applied to voltammetric analysis due to their high mechanical resistance, high chemical stability, low cost, low toxicity, and high reproducibility. Owing to those properties, PGEs found applications in different fields, such as environmental analysis, pharmaceutical and clinical analysis, food component, and contaminant analysis [141].

Pre-treatment or chemical modification of the surface of the electrodes leads to the improved electrochemical performance of PGEs towards pesticide detection, as discussed for GCEs, CPEs, and SPEs. For example, a film consisting of ionic liquid (IL), chitosan and electrochemically synthesised Au NPs were used as a modifier of the PGE for detecting the malathion pesticide [144]. The film consisted of separated graphite flakes and a uniform structure with Au NPs of an average diameter of about 50 nm. The EIS study confirmed a higher electron transfer rate for the modified electrode than bare PGE, which was attributed to the high conductivity of the composite and its large surface area. The sensor had narrow linear concentration ranges in an nM range, i.e., 0.89–5.94 nM and 5.94–44.6 nM, with a low LOD of 0.68 nM. A much wider concentration range, i.e., 5 nM–1.1 µM, and LOD of 1.3 nM, was obtained for the PGE modified by porous-walled polypropylene hollow fibres (HF), covered with CuO NPs, MWCNTs and IL 1-butyl-3-methylimidazolium tetrafluorophosphate (BMIMPF_6_) [145]. In this study, the authors showed that the presence of each component improved the extraction efficiency and accumulation of the herbicide at the electrode’s surface.

One of the rare studies that focused on the simultaneous determination of four different species found regularly in river waters was performed recently [146]. A Pd NPs-modified PGE sensor was developed for the determination of the dye Direct Yellow 50, amino acid Tryptophan, caffeine, and CBM pesticide, respectively. When all four species were analysed simultaneously, the sensor showed a linear concentration range of 0.2–1.6 µM for the CBM pesticide, an LOD of 1.8·10^−8^ M, and no interferences between the analysed species, which shows the promising importance of PGE sensors for applications in environmental analysis.

Gold electrodes and their modification were also utilised in non-enzymatic electrochemical sensing of pesticides. For example, Au atomic clusters with a size of 0.5–2 mm were prepared as a modifier on a polycrystalline gold electrode for MP pesticide detection [147]. The enhanced activity of Au atomic clusters towards the reduction of MP was ascribed to their high surface-to-volume ratio, providing many binding sites accessible for catalysis and sensing. The method exhibited linearity in the nM- and µM-ranges, i.e., 1–10 nM and 10–80 µM, with a low LOD of 0.65 nM. Ag-doped Fe_3_O_4_ NPs [148] and rGO [149] were also used as modifiers of gold electrodes for the detection of methomyl and propamocarb pesticides, respectively.

Nanostructured self-assembled films deposited onto glass substrates were recently studied for non-enzymatic electrochemical sensing of pesticides. The nanocomposite, consisting of polypyrrole (PPy) and MWCNTs, was deposited onto the glass substrates covered with indium tin oxide (ITO) [150]. PPy-ITO- and PPy-MWCNTs-ITO-based sensors for the detection of diuron had the same linear concentration range and similar LOD values. However, the introduction of MWCNTs into the nanocomposite modifier contributed to the enhanced sensitivity of the sensor, which increased from 0.022 µA µM^−1^ for the PPy-ITO to 0.231 µA µM^−1^ for the PPy-MWCNTs-ITO sensor, respectively.

The implementation of MOFs into non-enzymatic electrochemical sensors for the detection of pesticides enables feasible/convenient and green preparation of the sensors on different substrates, enhancing the analytical performance greatly. The large specific area and hierarchical pores of carefully designed copper benzene-1,3,5-tricarboxylate (Cu-BTC) MOF increased the active area of the modified electrode significantly and, consequently, contributed to the enhanced adsorption capacity of the glyphosate pesticide when prepared on the ITO substrate [151]. The authors found a strong affinity of the chelating groups in the glyphosate to the Cu^2+^ of the prepared modifier. The peak current of the sensor decreased gradually in the stripping step with increasing concentration of the analyte, resulting in a wide overall linear concentration response in two ranges, i.e., 1.0·10^−12^–1.0·10^−9^ M and 1.0·10^−9^–1.0·10^−5^ M. The sensor also showed very low LOD of 1.4·10^−13^ M, good reproducibility and stability, which confirmed the importance of the MOFs’ utilisation in electrochemical sensing.

Stainless steel showed its application as a substrate in the non-enzymatic electrochemical analysis of pesticides when nanowall arrays of CaCO_3_-chitosan composite-film were grown on cathodic stainless steel (SS) foils by a facile one-step electrodeposition approach, and the as-modified working electrode was utilised in a sensor for detection of MP [152]. The film consisted of uniformly distributed freestanding nanowalls standing perpendicularly to the substrate. The nanowalls had a lateral dimension in micrometre size, with a height of approximately 500 nm and an average pore size of approximately 400 nm. The formation of such morphology was achieved using chitosan. The properties of chitosan, such as large surface area, open boundaries and interlaced porous-wall architecture, contributed significantly to the great electrochemical properties of the sensor (see Table 4).

Thin films of boron-doped diamond (BDD) have been recognised as excellent electrode materials in various applications, including the electrochemical analysis of pesticides. For example, França et al. [155] reported the individual determination of two pesticides, CBM and fenamiphos, and the studied sensor also showed application in the simultaneous determination of both pesticides, respectively. In another work, the BDD electrode was modified by a graphene layer, and the as-modified electrode was used to determine the other two kinds of pesticides simultaneously, CBR and paraquat. Interestingly, when compared to previous work on unmodified BDD [155], the graphene-modified BDD electrode exhibited very similar LOD values and very similar linear concentration ranges [156].

Nowadays, demands for the miniaturisation and flexibility of electrochemical sensors for on-site analysis are an ultimate trend in analytical chemistry, and, thus, a variety of designs have been proposed in the literature. For example, a flexible electrochemical sensor based on nanomaterial ink printed on-site for the detection of organophosphorus pesticides was proposed recently [157]. An electrode ink was fabricated by SiC and MWCNTs, which served as the working electrode. The ink was painted on the analyte sample surfaces and then stabilised by a layer of chitosan, which acted as a fixing glue. The studied electrode system exhibited two linear concentration ranges of 50–10,000 ng mL^−1^ and 2000–10,000 ng mL^−1^ and an LOD of 20 ng mL^−1^ for the ethyl parathion pesticide. Moreover, the evaporation of parathion on the electrode surface was followed by painting the ink directly on a sweet potato leaf.

**Figure 9:**
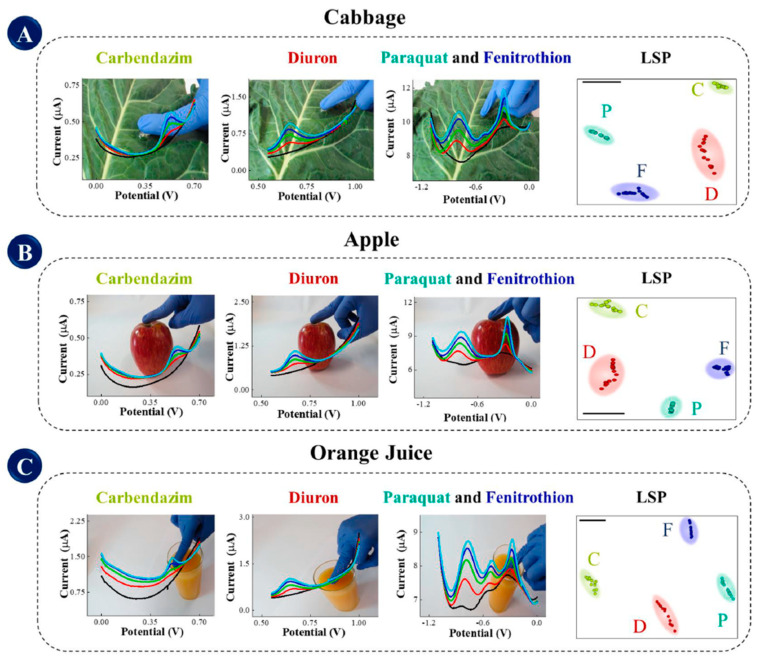
Demonstration of the measurements performed with glove-embedded sensors on real samples of cabbage (**A**), apple (**B**), and orange juice (**C**) for four pesticides, carbendazim, diuron, paraquat, and fenitrothion. Corresponding voltammograms are presented, including the results of simultaneous detection of paraquat and fenitrothion. The least square projection (LSP) plots present the measurements performed with the DPV and SWV techniques, where each coloured dot represents the corresponding voltammogram. Reprinted with permission from Ref. [158]. Copyright 2020 Elsevier B.V.

A set of three glove-embedded sensors was recently printed on three fingers of a rubber glove and studied as a flexible electrochemical sensor for detecting four different classes of pesticides, as shown in Figure 9 [158]. Carbon-based inks with different compositions were prepared to fabricate screen-printed electrodes on gloves, each composition on the selected finger. The carbon spherical shells (CSS) were used to detect CBM, while the Printex carbon nanoballs (PCN) were used to detect diuron. Moreover, the pretreated electrode was used successfully to detect paraquat and fenitrothion pesticides simultaneously. Such sensors showed good applicability in on-site monitoring of pesticides, with high selectivity for all four pesticides and good sensor reproducibility.

A miniaturised and robust electrochemical sensor using micropipette tips and metallic wires was designed to detect different electroactive species, including the CBM pesticide [159]. The working electrode, a Pt wire, was modified chemically with MWCNTs by a drop-casting procedure. The MWCNTs-Pt-based sensor showed a narrow linear concentration range (0.25–2.5 µM). However, high sensitivity and the application to real sample analysis of the newly designed device were observed.

## 3. Conclusions and Future Perspectives

This work shows a systematic overview of recent advances in the development and characterisation of the modified non-enzymatic electrochemical sensors used to determine and quantify pesticides. The main emphasis was the utilisation of nanomaterials as modifiers, and their influence on the analytical properties of the sensors for the selected electrode systems. The most frequently studied electrochemical system for applications in the non-enzymatic detection of pesticides was based on a glassy carbon electrode, which was characterised by good (electro)chemical stability and offered the possibility for simple modification of the surface by various deposition techniques. A variety of nanomaterials were developed and used as modifiers of the electrodes. The modifications were classified as single nanostructures, such as metal (or metal oxide) (e.g., Gd_2_O_3_, Au) NPs and carbon-based nanostructures (e.g., MWCNTs) in addition to nanocomposites. Among the latter, carbon-based nanostructures such as graphene(oxide) and CNTs are the most commonly used constituents of binary composites, in addition to some other metal(oxide) NPs and polymers. In the case of ternary nanocomposites, carbon-based materials, either (reduced) graphene oxide, carbon nanotubes, nanowires, or nanosheets, were reported in combination with metal or metal oxide (e.g., Au, Pt, ZnO, ZrO_2_, SiO_2_, TiO_2_, Fe_3_O_4_, and CuO) NPs and a conductive polymer (Nafion, polyaniline, carboxymethyl chitosan).

Carbon-based nanomaterials and metal or metal oxide NPs were the most frequently used modifiers of carbon paste electrodes and screen-printed electrodes, either in the shape of a single nanomaterial or as constituents of binary nanocomposites. Notably, other electrodes, such as graphite pencils, flexible on-site printed electrodes, and others, have recently attracted much attention due to their promising analytical properties achieved by the implementation of various nanomaterials such as Au NPs, Pd NPs, Au atomic clusters, graphene oxide, MWCNTs, chitosan nanowalls, etc.

In this work, different classes of pesticides were included, among which the most frequently analysed pesticide is the organophosphorus pesticide methyl parathion, followed by carbendazim, carbofuran, diuron and glyphosate and others. By comparing the analytical properties of the studied sensors, it was shown that, regardless of the type of the electrode system and the analyte, the modification of the electrode’s surface resulted in enhanced electrochemical performance of the sensors. Among all modifications, the utilisation of binary and ternary nanocomposites led to the most promising analytical properties of the studied sensors for real applications, such as low limit of detection, wide linear concentration range, high sensitivity and selectivity, repeatability, and others. These facts complementary prove the importance of nanocomposites implemented as modifiers into the electrochemical sensors with the desired analytical properties. However, it is not straightforward to predict the analytical properties of the sensors according to the type of modifier used for a specific electrode system. This still remains challenging in the field of the electroanalysis of pesticides and needs further research.

A literature survey revealed that the most common analysis of the electrochemical response performed by non-enzymatic sensors for the detection of pesticides is focused on the detection and quantification of the selected analyte, regardless of the type of electrode, the modifier and the analysed pesticide. These findings show the need to design and develop sensors that would enable the detection of multiple pesticides simultaneously, which would increase their use in real sample analysis applications. One of the possible approaches to overcome these deficiencies could involve the study of the electrode activation, i.e., pre-treatment of the electrode in acidic media, which also affects the electrochemical performance of the sensors. Another possibility, considering the application of nanocomposites in electrochemical sensing, could also involve the importance of 3D-printing technology as a new tool to fabricate portable nanocomposite-based sensors with the required analytical performance. The latter technology offers the development of electrochemical sensors with a wide range of designs and compositions, with the possibility of surface modification.

## Figures and Tables

**Table 1:** Summary of validation parameters for non-enzymatic electrochemical sensors using modified GCEs for pesticide detection. Repeatability is reported as RSD (in %) at a given concentration of the analyte. The recovery determined in real sample analysis is given from the minimum to the maximum values as reported.

Analyte	Modification	Supporting Electrolyte, pH	Detection Technique	LOD		LOQ		Linear Concentration Range	Sensitivity	Repeatability: RSD at Certain Concentration (%)	Special Observation (Real Sample Analysis, Interferences, …)	Recovery at Certain Concentration (%)	Ref.
				As Reported	Calculated (µM)	As Reported	Calculated (µM)						
**Glassy Carbon Electrode (GCE)**													
**Single Nanomaterial**													
CBM	MWCNT	0.1 M H_2_SO_4_, pH 1.0	DPSV	0.01 µg L^−1^	5.23·10^−5^	NR	/	0.01–5·10^4^ µg L^−1^	0.8326 µA µg L^−1^	2.3 (NR)	Real samples: soil, water, interferences: Cl^−^, Br^−^, SO_4_^2−^, NO_3_^−^, phenol, *o*-Chloro phenol, endosulfan, MP, malathion	82.10–93.73 (10–300 µg L^−1^)	[44]
MP	Gd_2_O_3_ hollow nanospheres	0.05 M phosphate buffer, pH 7	DPV	0.03 µM	3.00·10^−2^	NR	/	0.05–100 µM	0.1834 µA µM^−1^	NR	Real samples: cabbage, tap water, paddy field water, interferences: ascorbic acid, hydroquinone, glucose, M-nitrophenol, Imidacloprid, Pyrazosulfuron, 4-nitrobenzaldehyde, nitrobenzene, PO_4_^3−^, SO_4_^2−^, NO_3_^−^, Fe^2+^, Ni^2+^, K^+^	95.5–106 (1–5 µM)	[45]
MP	Nanoporous Au	100 mM HAC-NaAC solution, pH 4.0	DPV	0.02 µM	2.00·10^−2^	NR	/	0.5–150 µM	186.53 µA mM^−1^ cm^−2^	NR	NR	NR	[46]
CBM				0.24 μM	24.00·10^−2^	NR	/	3.0–120 µM	484.51 µA mM^−1^ cm^−2^	NR	NR	NR	[46]
MP and CBM simultaneously				0.085 μM (MP) 0.27 μM (CBM)	8.50·10^−2^ (MP) 27.00·10^−2^ (CBM)	NR	/	3–25 μM (MP) 10–70 μM (CBM)	629.68 µA mM^−1^ cm^−2^ (MP) 20.53 µA mM^−1^ cm^−2^ (CBM)	<2.6 (20 µM MP, 20 µM CBM)	Real sample: wastewater and seawater, interferences: Mg^2+^, K^+^, Na^+^, NH_4_^+^, SO_4_^2−^, PO_4_^3−^, CO_3_^2−^, NO_3_^−^, thiabendazole, methomyl, chlorpyrifos, tebuconazole, benomyl	94.93–104.73 (3.0–25.0 μM MP) 94.92–103.48 (10.0–70.0 μM CBM)	[46]
**Binary Nanocomposites**													
MP	MoS_2_-graphene NS	0.1 M phosphate buffer, pH 7	amperometry	3.23 µM	3.23	NR	/	10 nM–1.9 mM	0.457 µA µM^−1^ cm^−2^	3.9 (200 µM)	Real samples: apple, kiwi, tomato, cabbage, interferences: Cl^−^, I^−^, Zn^2+^, NO_3_^2−^, Cu^2+^, Ba^2+^, Ca^2+^, dopamine, uric acid, ascorbic acid, glucose, diuron, fenuron, SO_4_^2−^, NO_3_^2−^, nitrobenzene, 4-nitrophenol, 2-aminophenol, 4-aminophenol, 4-nitroaniline, 4-acetamidophenol and chloramphenicol	NR	[47]
Malathion	CuO NP-3D graphene	0.1 M Na_2_HPO_4_-citrate buffer, pH 5	DPV	0.01 nM	1.00·10^−5^	NR	/	0.03–1.5 nM	31.96%/nM	3.25 (at 1 nM)	Real sample: lake water, interferences: Na^+^, K^+^, Ca^2+^, Mg^2+^, Zn^2+^, Cl^−^, NO_3_^−^, PO_4_^3−^, SO_4_^2−^, glucose, carbentazim, lindane, trichlorphon	95.4–102.4 (at 0.3–1.5 nM)	[48]
Paraoxon ethyl	Graphene-NiFeSP	0.1 M phosphate buffer, pH 7	SWV	3.7 nmol L^−1^	3.70·10^−3^	NR	/	0.01–1.00 µM and 1.00–10.00 µM	10.243 µA L µmol^−1^ and 2.6267 µA L µmol^−1^	5.2 (at 8.0 μmol L^−1^)	Real samples: tap water, tomato juice, cucumber juice, interferences: PO_4_^3−^, SO_4_^2−^, NO_3_^−^, 4-nitrophenol, carbaryl, fenamiphos, MP	98–102.3 (at 150–1000 nmol L^−1^)	[49]
Diazinon	CNTs-TiO_2_	0.05 M phosphate buffer, pH 7	SWV	3 nM	3.00·10^−3^	10 nM	10.00·10^−3^	11–8360 nM	1.1753 µA µM^−1^	3.8 (NR)	Real samples: agricultural well water, city piped water	97.5–105.5 (at 1.0–2.0 µM)	[50]
Profenofos	3D CNTs-MIP	0.1 M phosphate buffer, pH 7	amperometry	0.002 μM	2.00·10^−3^	0.007 μM	0.007	0.01–200 μM	0.573 µA µM^−1^	4.8 (at 0.5 μM)	Real samples: Spring onion, tomato, Chinese cabbage, cabbage, green pepper, chili pepper, interferences: chlorpyrifos, carbofuran, hydroquinone, caffeine, phenol, MgSO_4_, NaCl	100.1–105.4 (at 0.05–0.1 μM)	[51]
Dicapthon	SWCNTs-Nafion	0.01 M B-R buffer, pH 5.0	DPV	0.036 µg L^−1^	1.21·10^−4^	0.054 µg L^−1^	1.81·10^−4^	0.2–60 µg mL^−1^	0.8535 µA cm^−2^ µg^−1^ mL	3.2 (NR)	Real samples: tap and well water, rice, corn, interferences: Pb^2+^, Cd^2+^, Mn^2+^, Cu^2+^, Co^2+^, Fe^2+^, Zn^2+^, Ca^2+^, Mg^2+^, ascorbic acid, dopamine	98.00–99.50 (at 10–40 µg mL^−1^)	[52]
Nitenpyram	HMWCNT-CNH	0.1 M phosphate buffer, pH 11	DPV	4.0 nM	4.00·10^−3^	NR	/	20–2000 nM	0.0158 µA nM^−1^	5.19 (at 1000 nM)	Real samples: corn, river water, interferences: ascorbic acid, fipronil, glucose, vitamin A	93.41–109.73 (at 20–200 nM)	[53]
CBM	CMC-MWCNT	0.1 M phosphate buffer, pH 7.0	DPV	0.015 µM	15.00·10^−3^	NR	/	0.03–10 µM	6.588 µA µM^−1^	1.68 (NR)	Real samples: peer and kiwifruit, interferences: Na^+^, Cl^−^, K^+^, NO^−3^, fructose, sucrose	97.67–100.5 (at 1.000–4.000 μM)	[54]
CBM	MoS_2_ QD-MWCNTs	0.1 M phosphate buffer, pH 7.0	SWV	0.026 µM	26.00·10^−3^	NR	/	0.04–1.00 µM	12.0171 µA µM^−1^	NR	Real samples: platycodon grandiflorum, pears, interferences: MgCl_2_, CaCl_2_, KCl, Pb(NO_3_)_2_, ascorbic acid, carotene	97.31−105.57 (at 0.3–1.0 µM)	[55]
CBM	SiO_2_-MWCNT	0.1 M phosphate buffer, pH 8.0	SWV	0.056 µM	56.00·10^−3^	0.187 µM	0.187	0.2–4.0 μM	0.485 A mol L^−1^	1.4 (at 2.0 μM)	Real samples: commercial orange juice, interferences: methomyl, carbaryl, ascorbic acid, citric acid	94.6–104 (at 0.5–5.0 μM)	[56]
CBM	Nd_2_Mo_3_O_9_-MWCNTs	0.1 M phosphoric acid buffer, pH 7.0	DPV	0.0167 nM	1.67·10^−5^	NR	/	5.0·10^−5^–9.0 µM	6.227 µA µmol L^−1^	NR	Real sample: water, interferences: Na^+^, K^+^, NH^4+^, Cu^2+^, Cd^2+^, Al^3+^, Cl^−^, CO_3_^2−^, SO_4_^2+^, PO_4_^3−^_,_ MP, fenitrothion, malathion, dichlorophenol, benomyl, thiabendazole, thiophanate, thiophanate-methyl, fuberidazole, glucose, ascorbic acid, vitamin B, C, E, dopamine, serine	96.7–102.0 (at 0.006–8.00 µM)	[57]
MP	Acetylene black-chitosan	Mcllvaine buffer, pH 5.6	DPV	2·10^−9^ mol L^−1^	2.00·10^−3^	NR	/	2·10^−8^–1·10^−4^ M	0.2528 µA L/µmol	1.49 (at 1·10^−5^ M)	Real sample: cabbage, interferences: Na^+^, K^+^, Ca^2+^, Mg^2+^, Cu^2+^, Cl^−^, NO_3_^−^, PO_4_^3−^, SO_4_^2−^, CO_3_^2−^, amino acid, glucose, sucrose, malathion, ascorbic acid, uric acid, p-aminophenol, o-, m- and p-phenylenediamine, nitrobenzene	95.4–105.1 (at 0.8–2.0 µM)	[58]
Malathion	CuO-CeO_2_	0.1 M phosphate buffer, pH 5.0	DPV	3.3 fM	3.00·10^−9^	NR	/	10 fM–100 nM	2.07 μA nM^−1^ cm^−2^	3.9 (at 1 nM)	Real samples: lake water, garlic, apple, interferences: chlorpyrifos, parathion, paraoxon, malaoxon, carberidazim, thiabendazole, cysteine, glutathione, mercaptoethanol, glucose, nitrobenzene, nitrophenol, Na^+^, K^+^, Fe^2+^, Fe^3+^, Al^3+^, Cl^−^, NO_3_^−^, SO_4_^2−^, PO_4_^3−^	96.2– 103.5 (at 0.02–1.8 nM)	[59]
Carbaryl	GO-[Bmim]PF_6_	B-R buffer, (pH 5.0)-methanol-water	SWV	0.02 µM	0.02	NR	/	0.10–12 µM	1.1 µA µM^−1^	3.2 (at 2 µM)	Real sample: grape, tomato, interferences: K_2_SO_4_, MgCl_2_, Ca(NO_3_)_2_, hydroquinone, guanine, phenol, catechol, glucose, ascorbic acid	90.0–96.7 (at 0.5–1.5 µM)	[60]
Chlorpyrifos	TiO_2_-cellulose acetate	0.05 M tetra-n-butyl ammoniumbromide in methanol/water	CV	4.4 µM	4.40	14.7 µM	14.7	10–30 µM	NR	2.54 (at 50 µM)	Real samples: tap water, commercial sample, soil, interferences: other pesticides: MP, fenitrothion, chlorophenol, chloroaniline, chlorobenzene, Ca^2+^, Mg^2+^, Na^+^, NH_4_^+^, K^+^	91.84 (at 100 µM)	[61]
			DPV	3.5 µM	3.50	11.7 µM	11.7	20–110 µM	NR	NR	Real samples: tap water, commercial sample, soil	96.28 (at 100 µM)	
			amperometry	11.8 µM	11.80	39.2 µM	39.2	20–340 µM	NR	NR	Real samples: tap water, commercial sample, soil	96.46 (at 100 µM)	
Clomazone	Pt NPs-MWCNTs	0.1 M phosphate buffer, pH 7.0	DPASV	0.38 ng cm^−3^	1.59·10^−3^	0.61 ng cm^−3^	2.54·10^−3^	0.61–20.56 ng cm^−3^	1.09 nA ng^−1^ mL	NR	Interferences: Ca^2+^, Na^+^, Ag^+^, K^+^, Cl^−^, HCO_3_^−^, CO_3_^2−^, NO_3_^−^, linuron, imidacloprid, tebufenozide	NR	[62]
Glyphosate	MWCNT-CuPc	0.1 M phosphate buffer, pH 7.4	DPV	12.2 nmol L^−1^	12.2·10^−3^	NR	/	0.83–9.90 µM	6.14 µA cm^−2^ µM^−1^	NR	NR	NR	[63]
Dichlorodiphenyltrichloroethane	PDA-Fe_3_O_4_-MIP NPs	5.5 mM [Fe(CN)_6_]^3−^ 0.1 M KCl	EIS	6·10^−12^ M	6.00·10^−6^	NR	/	1·10^−11^–1·10^−3^ M	19.33 Ω pmol^−1^ L	3.28 (at 1·10^−3^ M)	Real sample: radish juice, interferences: tetrabromobisphenol A, 3,4-dihydroxybenzoic acid, hydroquinone solution, p-methoxychlor	89–102 (at 0.01–100 µM)	[64]
Paraoxon	Stearic acid-nanosilver	Phosphate buffer, pH 7	DPV	0.1 nM	0.10·10^−3^	NR	/	0.1–5 nM	NR	2.7 (at 50 µM)	Real samples: onion, paddy grains, interferences: Na^+^, Ca^2+^, Mg^2+^, Fe^2+^, NH_4_^+^, K^+^, lindane, chlorpyrifos, imidacloprid, fenitrothion, thiamethoxam, monocrotophos, malathion	100.00 (at 0.2–0.5 nM)	[65]
Carbofuran (CBF) and carbaryl (CBR) simultaneously	35MIL(Fe)-101-rGO	0.1 M B-R buffer/ Acetonitrile, pH 4.0	DPV	0.52 nM (CBF) 0.11 nM (CBR)	0.52·10^−3^ (CBF)0.11·10^−3^ (CBR)	NR	/	5.0–200.0 nM (CBF) 1.0–300.0 nM (CBR)	0.1286 µA nM^−1^ (CBF) 0.0895 µA nM^−1^ (CBR)	2.9 CBF 3.2 CBR (at 100 nM CBF and CBR)	Real samples: cucumber, tomatoes, oranges, cabbages, interferences: Co^2+^, Ni^2+^, Cu^2+^, Cd^2+^, K^+^, Ca^2+^, Mg^2+^, Fe^3+^, Al^3+^, Ni^2+^, Zn^2+^, Cu^2+^, F^−^, Cl^−^, Br^−^, SO_4_^2−^, PO_4_^3−^, NO_3_^−^, CO_3_^2−^, diazinon, malathion, paraoxon, parathion, fenamiphos	98.0–104.7 (at 100–160 nM)	[66]
**Ternary Nanocomposites**													
Imidacloprid	ZnO-PANI-GO	0.1 M phosphate buffer, pH 5.8	CV	1.3·10^−8^ M	1.30·10^−2^	1.3·10^−7^ M	0.13	1.25·10^−7^–2.12·10^−6^ M	1.5604 A M^−1^	NR	Real samples: chilli, tomato, potato	98.23–104.37 (at 1.00·10^−6^–1.75·10^−6^ M)	[67]
MP	MnO_2_-PTH-rGO	0.1 M phosphate buffer, pH 7.0	amperometry	5.72 nM	5.72·10^−3^	NR	/	10 nM–1 µM	0.0498 µA µM^−1^ cm^−2^	NR	Real samples: human urine and serum	88.5–97.2 (at 0.5–10 µM)	[68]
MP	Au-ZrO_2_-GNS	0.1 M phosphate buffer, pH 5.6	SWV	1 ng mL^−1^	3.80·10^−3^	NR	/	1–100 ng mL^−1^ and 100–2400 ng mL^−1^	0.00351 µA ng^−1^ mL and 0.01136 µA ng^−1^ mL	NR	Real sample: chinese cabbage, interferences: p-nitrophenol, p-nitroaniline, trinitrotoluene, NO_3_^−^, PO_4_^3−^, SO_4_^2−^	96.2–102.1 (at 300–1500 ng mL^−1)^	[69]
MP	Au NP-chitosan-GNS	0.1 M phosphate buffer, pH 5.7	SWASV	0.6 ng mL^−1^	2.28·10^−3^	NR	/	0.001–0.1 and 0.2–1.0 µg mL^−1^	256.3 µA µg^−1^ mL and 11.7 µA µg^−1^ mL	5.6 (at 0.1 µg mL^−1^)	Real samples: garlic, cabbage, tea, interferences: as p-nitrophenol, nitrobenzene, p-nitroaniline, trinitrotoluene, PO_4_^3−^, SO_4_^2−^, NO_3_^−^	96.2–105 (at 5.86–6.17 ng mL^−1^)	[70]
MP	Pd-MWCNTs-Nafion	0.1 M phosphate buffer, pH 7.0	DPV	0.05 μg mL^−1^	19.00·10^−2^	NR	/	0.10–14 μg mL^−1^	18.30 µA μg^−1^ mL	4.6 (at 2.0 μg mL^−1^)	Interferences: Cl^−^, PO_4_^3−^, SO_4_^2−^ and NO_3_^−^	NR	[71]
Fenitrothion	SiO_2_-MWCNTs-RuPc	0.1 M acetate buffer, pH 4.5	DPV	1.62 µM	1.62	NR	/	3·10^−6^–6·10^−5^ M	0.0822 µA µmol^−1^ L	2.3 (at 16.6 µmol L^−1^)	Real sample: fresh orange juice, interferences: malathion, chlorpyrifos, ascorbic acid	91.6–98.8 (at 6.10–24.98 µmol L^−1^)	[72]
CBM	CMC-MWCNTs-MoS_2_	0.1 M phosphate buffer, pH 7.0	DPV	7.4 nM	74.00 10^−2^	NR	/	0.04–9 µM	NR	0.57 (NR)	Real samples: tea, rice, interferences: vitamin C, vitamin B2, imidacloprid, glyphosate, endosulfan, buprofezin, fructose, sucrose, L-arginine, L-serine	89.18–105.56 (0.45–4.2 µM)	[73]
CBM	Fullerene-MWCNTs-Nafion	0.1 M ammoniacal buffer, pH 9	SWV	1.7·10^−8^ M	1.70·10^−2^	5.57·10^−8^	5.57·10^−2^	2·10^−8^–3.5·10^−7^ M	419.69 A mol^−1^ L	3.12 (at 5·10^−7^ M)	Real sample: soil, interferences: K^+^, Na^+^, Ca^2+^, Mg^2+^, Fe^3+^	37.8–38.4 (at 5·10^−5^ M)	[74]
CBM	IL-CaFe_2_O_4_-MWCNTs	0.2 M phosphate buffer, pH 4.0	DPV	9.41 nM	9.41·10^−3^	NR	/	3.14·10^−8^–1.05·10^−5^ M and1.05·10^−5^–1.05·10^−4^ M	2.009 µA µmol^−1^ L and 0.297 µA µmol^−1^ L	3.5 (at 5.23·10^−5^ M)	Real samples: paddy water, apple, tomato, interferences: K^+^, Na^+^, Mg^2+^, Zn^2+^, Ni^2+^, PO4^3−,^ Cl^−^, NO^3 −^, CO_3_^2−^, HCO_3_^2−^, SO_4_^2−^, thiabendazole, tricyclazole, pyrimethanil, paranitrophenol	94.7–105.5 (at 4.18·10^−6^–7.23·10^−5^ M)	[75]
Paraoxon ethyl	Au-ZrO_2_-SiO_2_	0.2 M acetate buffer, pH 5.2	SWV	0.5 ng mL ^1^	1.82·10^−3^	NR	/	1.0–500 ng mL^−1^	NR	NR	Interferences: nitrobenzene, nitrophenol, PO_4_^3−^, SO_4_^2−^, NO_3_^−^	NR	[76]
MP	CuO-TiO_2_-Nafion	0.1 M phosphate buffer, pH 6	DPV	1.21 ppb	4.60·10^−3^	NR	/	10–500 ppb	0.0412% ppb^−1^	2.9 (at 10 ppb)	Real sample: ground water, interferences: trichlorphon, caeberidazim, carbaryl, 4-nitrobenzaldehyde, nitrobenzene, PO_4_^3−^, SO_4_^2−^, NO_3_^−^, Fe^2+^, Ni^2+^, K^+^	98.80–106.20 (at 40–200 ppb)	[77]
Methomyl	Ag-Fe_3_O_4_-chitosan	0.2 M phosphate buffer, pH 6.9	CV	2.97·10^−5^ M	29.70	NR	/	3.47·10^−5^–3.47·10^−4^ M	0.009166 A mol^−1^ L	NR	Real sample: lettuce, rape, spinach, interferences:	93.08–96.45 (at 0.0121–0.0325 mg·kg^−1^)	[78]
CBM and thiabendazole (TBZ) simultaneously	ZnFe_2_O_4_-SWCNTs-Nafion	0.2 M phosphate-buffered saline, pH 7.0 + 10.0 µg/mL CTAB	DPV	0.09 µM (CBM) 0.05 µM (TBZ)	9.00·10^−2^ (CBM) 5.00·10^−2^ (TBZ)	NR	/	1.0–100.0 µM (CBM) 1.0–100.0 µM (TBZ)	1.039 µA µmol L^−1^ (CBM) 0.798 µA µmol L^−1^ (TBZ)	NR	Real samples: apple, leek, tomato, paddy water, sea water, interferences: Na^+^, K^+^, NH^4+^, Cl^−^, NO_3_^−^, H_2_PO_4_^−^, HCO_3_^−^, CO_3_^2−^, SO_4_^2−^, Mg^2+^, Pb^2+^, Cu^2+^, Zn^2+^, Cd^2+^, ascorbic acid, catechin, anthocyanin, triadimenol, tricyclazole, paranitrophenol, Pyrimethanil	88.2–104.4 (at 5.0–50.0 µM)	[79]
Carbofuran (CBF) and carbaryl (CBR) simultaneously	CoO-rGO-Nafion	0.1 M B-R buffer/acetonitrile, pH 4	DPV	4.2 μg/L (CBF) 7.5 μg/L	1.90·10^−2^ (CBF) 3.73·10^−2^ (CBR)	NR	/	0.2–70 µM (CBF) 0.5–200 µM (CBR)	0.07045 µA cm^2^/µM (CBF) 0.01952 µA cm^2^/µM (CBR)	2.9 (at 30 µM CBF, 70 µM CBR)	Real samples: grapes, oranges, tomato, cabbages, interferences: Na^+^, K^+^, Mg^2+^, Ca^2+^, Zn^2+^, Al^3+^, F^−^, Cl^−^, CO_3_^2−^, SO_4_^2−^, NO^−^, isoprocarb, methiocarb, propoxur, hydroquinone, xanthine, guanine, phenol, catechol, caffeine.	96.0–104.0 (at 0.50–1.00 µM CBF) 96.6–102.6 (at 5.00–10.00 µM CBR)	[80]
Paraoxon and chlorpyrifossimultaneously	TiO_2_-GO-UiO-66	0.1 M B-R buffer/ acetonitrile, pH 5	SWV	0.22 nM (paraoxon) 1.20 nM (chlorp.)	2.20·10^−4^ (paraoxon) 1.20·10^−3^ (chlorp.)	NR	/	1.0–100.0 nM (paraoxon) 5.0–300.0 nM (chlorpyrifos)	0.3393 µA nM^−1^ (paraoxon) 0.091 µA nM^−1^ (chlorpyrifos)	2.6 (at 50 nM) (paraoxon) 2.2 (at 50 nM) (chlorpyrifos)	Real samples: tap water, celery, lettuce, cabbage, interferences:Cl^−^, SO_4_^2−^, CO_3_^2−^, NO_3_^−^, PO_4_^3−^, Cu^2+^, Zn^2+^, Pb^2+^, Fe^2+^, Cd^2+^	97.0–106.4 (at 50–70 nM)	[81]
**Multiple Nanocomposites**													
Carbofuran	MAA-EGMRA-ABIN-rGO-Au NP	0.1 M KCl, pH 7.0	DPV	0.02 µM	2.00·10^−2^	NR	/	5·10^−8^–2·10^−5^ M	0.04917 µA µmol^−1^ L	1.1 (at 1.0·10^−7^ M)	Real samples: cabbage, cucumber, inferferences: carbaryl, metolcarb, 3,5-xylyl methylcarbamate	97.7–110.6 (at 1–20·10^−6^ M)	[82]
Trichlorfon	MWCNT-TiO_2_-CMCh-Nafion	0.2 M phosphate buffer, pH 7.0	DPV	4·10^−7^ M	40.00·10^−2^	NR	/	1·10^−11^–1·10^−5^ M	0.5077 µA µM^−1^	1.57 (at 1·10^−6^ M)	Real samples: apple, mushroom, cucumber	72.0–98.0 (at 0.5–4.0·10^−10^ M)	[83]

**Table 2:** Data of recently reported electrochemical sensors for non-enzymatic pesticide detection using modified carbon paste electrodes (CPEs).

Analyte	Modifier	Supporting Electrolyte, pH	Detection Technique	LOD		LOQ		Linear Concentration Range	Sensitivity	Repeatability: RSD at Certain Concentration (%)	Special Observation (Real Sample Analysis, Interferences, …)	Recovery at Certain Concentration (%)	Ref.
				As Reported	Calculated (µM)	As Reported	Calculated (µM)						
**Carbon Paste Electrodes (CPEs)**													
Glyphosate	None	0.2 M B-R buffer, pH 5.0	SWV	2.0 nM	2.0·10^−3^	7.0 nM	7.00·10^−3^	4.40·10^−8^–2.80·10^−6^ M	27.14 μA µM^−1^	NR	Real samples: orange juice, milk and agricultural formulations, interferences: Na^+^, NH_4_^+^, Ca^2+^, Mg^2+^, Al^3+^, Cu^2+^, Cl^−^, OH^−^, NO_3_^−^, SO_4_^2−^, atrazine, linuron, thiamethoxam, trifluralin, dichlorophenoxyacetic acid, trifloxystrobin, ascorbic acid	98.31–103.75 (at 21.10–84.40 nM)	[100]
Fenhexamid	None	0.1 M B-R buffer, pH 4, 10 vol.% MeOH	SWV	0.97 µM	0.97	NR	/	3.22–44.60 µM	0.120 μA µM^−1^	NR	Real samples: blueberries, strawberries, red wine grapes, white wine grapes	92.9–99.8 (at 5–50 µM)	[101]
CBM	None	0.1 M C_6_H_8_O_7_-Na_2_HPO_4_ buffer, pH 5.0	DPV	0.96 µg L^−1^	5.02·10^−3^	NR	/	2.84–45.44 µg L^−1^	0.101 μA L µg^−1^	1.05 (at 2.84 µg L^−1^)	Real sample: water, orange juice, interferences: orange juice, CuSO_4_, glyphosate, thiamethoxam, endosulfan	99.12–101.41 (at 2.84–22.72 µg L^−1^)	[102]
Linuron	None	0.2 M B-R buffer, pH 5.5	SWV	23.00 µg L^−1^	9.23·10^−2^	NR	/	25.75–309.02 µg L^−1^	0.01627 A L µg^−1^	NR	Real sample: natural water, distilled water, carrot, potato, onion, interferences	96.00–103.00 (at 50.25–59.80 µg L^−1^)	[103]
**Single Nanomaterial**													
Diazinon	MWCNTs	Acetate buffer, pH 5.25	DPV	4.5·10^−10^ M	4.50·10^−4^	NR	/	1·10^−10^–6·10^−8^ M	18.973 μA µM^−1^	NR	Real samples: tomato, apple, cucumber, spinach, sweet peppers, lettuce, cabbage, eggplant, interferences: K^+^, Ca^2+^, Mg^2+^, Ni^2+^	NR	[104]
Cyromazine	MWCNTs	0.1 M H_2_SO_4_	SWV	0.12 µg mL^−1^	7.22·10^−1^	0.41 µg mL^−1^	2.47	0.41–83.30 µg mL^−1^	2.26 µA mL µg^−1^	NR	Real samples: river and tap water, agrochemical pesticide formulation Trigard^®^, interferences: Zn^2+^, Mg^2+^, Ni^2+^, Co^2+^, Na^+^, Cl^−^, Cu^2+^, Pb^2+^, cyanazine, atrazine, cymoxanil	96.7–101.5 (at 5.0–25.0 µg mL^−1^)	[105]
Fenhexamid	MWCNTs	0.1 M B-R buffer, pH 4, 10 vol.% MeOH	SWV	0.52 µM	52.00·10^−2^	NR	/	1.74–157.48 µM	0.108μA µM^−1^	NR	NR	NR	[101]
Cypermethrin	TiO_2_ NP	Citrate buffer, pH 5	DPV	0.0978 ppm	24.00·10^−2^	NR	/	0.1–1 ppm	8.4865 µA cm^−2^ ppm^−1^	0.37 (at 1 ppm)	NR	NR	[106]
MP	ZrO_2_ NP	Acetate buffer, pH 5.0	SWV	2 ng mL^−1^	7.60·10^−3^	5 ng mL^−1^	1.90·10^−3^	5–3000 ng mL^−1^	1.3461 µA mL µg^−1^	4.5 (at 0.050 µg mL^−1^)	Real samples: tap and river water, interferences: Na^+^, K^+^, NH_4_^+^, SO_4_^2−^, NO_3_^−^, Cl^−^, Ca^2+^, Mg^2+^, Ni^2+^, Co^2+^, Fe^2+^, Fe^3+^, Hg^2+^, Cr^3+^, Pb^2+^, Cd^2+^, Cu^2+^, nitrophenol, phenol	94.0–102.0 (at 0.050–0.800 µg mL^−1^)	[107]
Chlorpyrifos	Fe_3_O_4_	0.1 M phosphate buffer, pH 7.5	DPV	2.8·10^−6^ M	2.80	NR	/	1–100 µM	0.587 μA μM^−1^	3.42 (at 2.5 mM)	NR	92.9–99.8 (at 5–50 µM)	[108]
CBM	Ce-ZnWO_4_	0.1 M phosphate buffer, pH 7.0	DPV	0.003 μM	3.00·10^−3^	NR	/	0.01–~5.5 μM	3.5781 μA μM^−1^	±5 (at 5.0·10^−5^ M)	Real samples: dopamine, uric acid	(at 5.0·10^−5^ M)	[109]
CBM	La-Nd_2_O_3_	0.1 M phosphate buffer, pH 7.0	DPV	0.027 µM	0.27·10^−2^	NR	/	0.08–15 µM 15–50 µM	2.1760 μA μM^−1^ 0.8466 μA μM^−1^	2.94 (at 5 µM)	Interferences: NaCl, Mg(NO_3_)_2_, CuSO_4_, glucose, sucrose, ascorbic acid, pheno	NR	[110]
CBM	1-hexyl-3-methylimidazolium bis(trifluoromethylsulfonyl)imide	0.1 M B-R buffer, pH 5.0	DPASV	1.7 µg L^−1^	8.89·10^−3^	5.7 µg L^−1^	2.98·10^−2^	0.010–0.247 mg L^−1^	NR	1.3 (at 0.010 mg L^−1^)	Real sample: tap water, interferences: linuron, imidacloprid, acetamiprid	104.1 (NR)	[111]
**Binary Nanocomposite**													
Diazinon	MIP-MWCNTs	0.1 M acetate buffer, pH 4.0	SWV	4.1·10^−10^ M	4.10·10^−4^	1·10^−9^ M	1.00·10^−3^	5·10^−10^–1·10^−6^ M	0.9418 µA nM^−1^ 0.0942 µA nM^−1^	3.16 (NR)	Real sample: urine, tap water, river water, interferences: coumachlor, dicloran, dichlrofention, dimethoate, Cd^2+^, Ca^2+^, Mg^2+^, Pb^2+^, NO_3_^−^	92.00–97.50 (at 20–2000 ng mL^−1^)	[112]
Dicloran	MIP-MWCNTs	0.04 M KCl pH 8.0	SWV	4.8·10^−10^ M	4.80·10^−4^	9.4·10^−10^ M	9.40·10^−10^	5·10^−9^–1·10^−6^ M	0.1055 µA nM^−1^	NR	Real samples: tap water, river water, urine, interferences: carbofuran, diazinon, dichlrofention, dimethoate	89.70–100.30 (at 20–2000ng mL^−1^)	[113]
Diuron	MIP-MWCNTs-COOH	0.1 M phosphate buffer, pH 8.0	SWV	9.0·10^−9^ M	9.00·10^−3^	NR	/	5.2·10^−8^–1.25·10^−6^ M	5.1·10^5^ µA M^−1^	NR	Real sample: river water, interferences: metribuzin, 2,4-D, CBF, CBM	96.1–99.5 (at 5.2·10^−8^ M)	[114]
Linuron	MWCNTs-ZnO	0.2 M phosphate buffer, pH 6.0	SWV	5.83·10^−9^ M	5.83·10^−3^	1.94·10^−8^ M	1.94·10^−2^	0.02–0.34 µM	2.4239 μA μM^−1^	NR (0.1 mM)	Real samples: black soil, lake soil, agricultural soil, brick soil, red soil, water (pond, dam, tap, reverse osmosis, lake), interferences: CaCl_2_, CuSO_4_, MnSO_4_, KNO_3_, FeSO_4_, ZnCl_2_	96.2–99.42 (at 0.1·10^−5^–1.0·10^−4^ M)	[115]
CBM	MWCNT-Ca-ZnO	0.2 M phosphate buffer, pH 7.0	SWV	4.68·10^−9^ M	4.68·10^−3^	1.75·10^−8^ M	1.75·10^−2^	0.01–0.45 µM	2.2776 μA μM^−1^	NR	Real samples: soil, water	81.0–96.2 (at 0.2·10^−5^ –1.0·10^−4^ M)	[116]
Fluometuron	FePc-MWCNT	B-R buffer, pH 6.0	DPV	69.8 µg L^−1^	3.01·10^−1^	233 µg L^−1^	1.00	0.40–15.0 mg L^−1^	4.596 µA mg^−1^ L	3.83 (at 0.75 mg L^−1^)	Real samples: tap water, commercial herbicide formulations, interferences: captan, halosulfuron methyl, monocrotophos, pencycuron, tolclofos-methyl, teflubenzuron pesticides, Cu^2+^, Fe^2+^, Pb^2+^, Zn^2+^	96.0 ± 2.7 (at 0.75 mg L^−1^)	[117]
Fipronil	FeO-TiO_2_	1.0 M MgSO_4_	CV	0.0012 μM	12.00·10^−4^	NR	/	1.0·10^−3^–1.0·10^−2^ µM	NR	0.17 (at 1 µM)	Interference: Cu^2+^	NR	[118]
Fipronil	Al-TiO_2_	0.1 M HCl and Na_2_SO_4_	CV	0.0164 µg L^−1^	3.75·10^−5^	NR	/	0.01–0.09 µg L^−1^	325 µA L µg^−1^	NR	Interferences: Cd^2+^, Pb^2+^	NR	[119]
Isoproturon	CuO-CNTs	0.5 M H_2_SO_4_	CV	5·10^−10^ M	5.00·10^−4^	1.5·10^−9^ M	1.50·10^−3^	1·10^−8^–1·10^−6^ M	1.328 A M^−1^	2.0 (at 9·10^−8^ M)	Real sample: tap water, interferences: linuron, propazine, tetrazine, metazachlore, chlordecone	96.4–101.7 (at 0.2–0.6 µM)	[120]
CBM	FS-Ag NPs	0.1 M phosphate buffer, pH 7.4	DPV	9.4·10^−10^ M	9.40·10^−4^	NR	/	5.0·10^−8^–3.0·10^−6^ M 3.0·10^−6^–1.0·10^−5^ M	7.001 μA μM^−1^ 1.895 μA μM^−1^	NR	Real samples: river water, tomatoes juice, commercial apple and orange juices	92.1–105.6 (at 1.5·10^−5^ and 3.0·10^−5^ M)	[121]
Amino-triazole	g-C_3_N_4_-CTAB	Phosphate buffer, pH 4.2	SWV	6.41·10^−8^ M	6.41·10^−2^	2.14·10^−7^ M	2.14·10^−1^	3.0·10^−7^–4.5·10^−5^ M	13.645 μA μM^−1^	NR (at 0.1 mM)	Real samples: black soil, lake soil, agricultural soil, brick soil, red soil, water (pond, dam, tap, reverse osmosis, lake), interferences: CaCl_2_, MgSO_4_, FeSO_4_, ZnCl_2_, KCl, NaCl	95.50–99.50 (at 0.1·10^−5^ and 0.2·10^−5^ M)	[122]
Linuron	g-C_3_N_4_-CTAB	Phosphate buffer, pH 4.2	SWV	2.47·10^−8^ M	2.47·10^−2^	8.23·10^−8^ M	8.23·10^−2^	1.2·10^−7^–3.0·10^−4^ M	6.7148 μA μM^−1^	NR (at 0.1 mM)	Real samples: black soil, lake soil, agricultural soil, brick soil, red soil, water (pond, dam, tap, reverse osmosis, lake), interferences: CaCl_2_, MgSO_4_, FeSO_4_, ZnCl_2_, KCl, NaCl	89.20–98.00 (at 0.4·10^−5^ and 0.5·10^−5^ M)	[122]

**Table 3:** Data of recently reported electrochemical sensors for non-enzymatic pesticide detection using modified screen-printed electrodes (SPEs).

Analyte	Modification	Supporting Electrolyte, pH	Detection Technique	LOD		LOQ		Linear Concentration Range	Sensitivity	RSD at Certain Concentration (%)	Special Observation (Real Sample Analysis, Interferences, …)	Recovery at Certain Concentration (%)	Ref.
				As Reported	Calculated (µM)	As Reported	Calculated (µM)						
**Screen-Printed Electrodes (SPEs)**													
**Single Nanomaterial**													
Carbaryl	Nano carbon black	MeOH:phosphate buffer, pH 7.0	DPV	4.8·10^−8^ M	4.80·10^−2^	NR	/	1.0·10^−7^–1.0·10^−4^ M	4.94·10^−1^ A M^−1^ cm^−2^	NR	Real samples: durum wheat, organic durum wheat, soft wheat, organic soft wheat, maize	78–102 (at 0.25–0.75 mg kg^−1^)	[127]
Isoprocarb				7.9·10^−8^ M	7.90·10^−2^	NR	/	1.0·10^−7^–1.0·10^−4^ M	3.98·10^−1^ A M^−1^ cm^−2^	NR			
Fenobucarb				8.0·10^−8^ M	8.00·10^−2^	NR	/	1.0·10^−7^–1.0·10^−4^ M	3.90·10^−1^ A M^−1^ cm^−2^	NR			
Carbofuran				4.9·10^−8^ M	4.90·10^−2^	NR	/	1.0·10^−7^–1.0·10^−4^ M	4.86·10^−1^ A M^−1^ cm^−2^	NR			
Parathion	NiO NPs	B-R buffer, pH 6	DPV	24 nmol L^−1^	2.40·10^−2^	NR	/	0.1–5 and 5–30 µmol L^−1^	0.51 µA µM and 0.24 µA µM	2.87 (at 20 µM) 3.54 (at 1.0 µM)	Real samples: tap water, urine, tomato juice, interferences: CaCl_2_, FeCl_3_, KI, NaNO_3_, Na_2_SO_4_, durspan, imidacloprid, p-nitrophenol	94–103 (at 1–2 µM)	[128]
CBM	MWCNT	0.04 M B-R buffer, pH 4.00	SWV	1.40·10^−8^ M	1.40·10^−2^	4.21·10^−8^ M	4.21·10^−2^	4.00·10^−8^–4.01·10^−7^ M	19.2 µA M^−1^	3.1 (at 3.05·10^−6^ M)	Real sample: orange juice	100–103.2 (at 15.6 ppb)	[129]
MP	GO nanoribbons	0.1 M phosphate buffer, pH 7.0	Amperometry	0.5 nM	0.50·10^−3^	NR	/	0.1–100 µM 100–2500 µM	1.804 µA µM cm^2^ 0.8587 µA µM cm^2^	3.95 (at 0.1 µM)	Real samples: ugli fruit, tomato, beetroot, broccoli, interferences: Ni^2+^, Cu^2+^, Mn^2+^, Zn^2+^, Ca^2+^, Ba^2+^, NO_3_^−^, malathion, 4-nitrophenol, nitrobenzene, aminophenol, 2-nitro aniline, 4-nitro aniline, 4-acetamidophenol	NR	[130]
Diazinon	PCL-chitosan nanofibers	0.1 M acetate buffer, pH 5.25	DPV	2.888 nM	2.88·10^−3^	NR	/	3–100 nM	0.2041 µA µM	3.12 (at 10 nM)	Real sample: tomato juice, interferences: Ca^2+^, K^+^, Mg^2+^, Ni^2+^	93.27–108.30 (at 20–60 nM)	[131]
Paraoxon	BiVO_4_	0.1 M phosphate buffer, pH 7.0	DPV	0.034 µM	3.40·10^−2^	0.115 µM	1.15·10^−1^	0.2–1.96 µM	0.345 μA μM^−1^ cm^−2^	NR	Real sample: river water, interferences: glucose, dopamine, urea, uric acid, Ca^2+^, Zn^2+^, Mg^2+^, Na^2+^	95.01–98.42 (at 1–5 µM)	[132]
Isoproturon (ISO) and CBM simultaneously	Graphene	1.0 M HClO_4_, pH 2	SWV	0.02 mg L^−1^ (ISO) 0.11 mg L^−1^ (CBM)	9.70·10^−2^ (ISO) 5.75·10^−1^ (CBM)	0.07 mg L^−1^ (ISO) 0.38 mg L^−1^ (CBM)	3.39·10^−1^ (ISO) 1.99 (CBM)	0.02–10.0 mg L^−1^ (ISO) 0.50–10.0 mg L^−1^ (CBM)	0.4294 µA L mg^−1^ (ISO) 0.2417 µA L mg^−1^ (CBM)	9.2 (at 0.02 mg L^−1^ ISO) 10 (at 0.50 mg L^−1^ CBM)	Real samples: river water, rice-field water, rice-field soil, tomatoes, lettuce, interferences: CN^−^, CO_3_^2^^−^, NO_3_^−^, PO_4_^3^^−^, SO_4_^2^^−^, Ca^2+^, Cd^2+^, Co^2+^, Cu^2+^, K^+^, Mg^2+^, Na^+^, Ni^2+^, Pb^2+^, Zr^4+^, Zn^2+^, disulfiram, thiram	77.9–107 (at 2.00 mg L^−1^ ISO and CBM)	[133]
**Binary Nanocomposites**													
MP	Ag NP- graphene nanoribbons	Phosphate buffer, pH 7.0	amperometry	0.5 nM	5.00·10^−4^	NR	/	0.005–2780 µM	0.5940 µA µM^−1^ cm^−2^	4.51 (at 100 nM)	Real samples: cabbage, green beans, strawberry, nectarine, interferences: Ca^2+^, Cu^2+^, Mn^2+^, Ba^2+^, Ni^2+^, Zn^2+^, NO_3_^−^, 4-Acetaminophenol, 4-Nitrophenol, 4-Nirobenzene, 4-Aminophenol, 2-Nitro aniline, 4-Nitro Aniline, 4-acetamido phenol.	NR	[134]
MP	GO NS-ZnO	0.1 M phosphate buffer, pH 7.0	DPV	1.23 nM	1.23·10^−3^	8.61 nM	8.61·10^−3^	0.03–669.65 μM	16.5237 μA μM^−1^ cm^−2^	3.75 (at 50 µM)	Real samples: apple, broccoflower, collard greens interferences: fenitrothion, ethyl parathaion, thiamethoxam, imidacloprid, catechol, hydroquinone, resorcinol, tannic acid, NaCl	98.00–98.50 (2–5 µM)	[135]
Methyl paraoxon	GO NS-CuFeS_2_	Phosphate buffer, pH 7	DPV	4.5 nM	4.50·10^−3^	NR	/	0.073–801.5 µM	17.97 µA µM^−1^ cm^−2^	3.72 (at 50 µM)	Real samples: lettuce, cherry tomato, interferences: 2,4 di-tert-butylphenol, fructose, butylated hydroxyl anisole, propylgallate, ascorbic acid, folic acid, Ca^2+^, glucose, caffeic acid	96.36–99.68 (at 10–20 µM)	[136]
Fenitrothion	NbC-Mo	0.1 M phosphate buffer, pH 7.0	DPV	0.15 nM	1.50·10^−4^	NR	/	0.01–1889 µM	0.355 µA µM^−1^ cm^−^^2^	3.23 (at 50 µM)	Real samples: grapes and cranberry extracts, interferences: ascorbic acid, catechol, glucose, caffeic acid, uric acid hydroquinone, dopamine, Ca^2+^, K^+^, Zn^2+^, Fe^2+^, Ba^2+^, Cu^2+^, NO_2_^−^, SO_4_^2−^, NO_3_^−^, I^−^, Br^−^, Cl^−^, urea, 4-nitrophenol, 4-nitrobenzene, fenamiphos, carbofuran, azathioprine	NR	[137]
CBF and CBM simultaneously	GO-CTAB	0.1 M phosphate buffer, pH 7	SWV	10 µg L^−1^ (CBF) 5 µg L^−1^ (CBM)	4.52·10^−2^ (CBF) 2.62·10^−2^ (CBM)	NR	/	40–20,000 µg L^−1^ (CBF) 25–5000 µg L^−1^ (CBM)	0.0003 µA L µg^−1^ (CBF) 0.002 µA L µg^−1^ (CBM)	NR	Real samples: soybeans, rice, tomatoes	95.7–105.5 (at 50–4000 CBF, CBM)	[138]
**Ternary Nanocomposites**													
CBM	Chitosan-fC-Cu	0.05 M phosphate buffer, pH 7.0	LSV	0.028 μM	2.80·10^−2^	NR	/	0.8–277.0 μM	0.0981 μA μM	NR	Real samples: environmental water, interferences: diuron, bentazon, diphenylamine, carbofuran	97.0–98.5 (5–40 µM)	[139]
MP	NiS_2_-rGO NS-curcumin NP	Phosphate buffer, pH 7.4	DPV	8.7 nM	8.70·10^−3^	NR	/	0.25–5 μM 5–80 μM	7.165 μA μM^−1^ cm^−2^ 2.796 μA μM^−1^ cm^−2^	2.1 (at 40 µM)	Real samples: tomato and apple juices, river water, interferences (investigated with AMP): dinotefuran, H_2_O_2_, tannic acid, NaSO_4_, catechol, hydroquinone, 2,4-dinitrobenzene	96.5–100.6 (at 20 µM)	[140]

**Table 4:** Data of recently reported electrochemical sensors for non-enzymatic pesticide detection using other types of electrodes.

Analyte	Modifier	Supporting Electrolyte, pH	Detection Technique	LOD		LOQ		Linear Concentration Range	Sensitivity	RSD at Certain Concentration (%)	Special Observation (Real Sample Analysis, Interferences, …)	Recovery at Certain Concentration (%)	Ref.
				As Reported	Calculated (µM)	As Reported	Calculated (µM)						
**Pencil Graphite Electrodes**													
Malathion	IL-chitosan-Au NP	0.2 M B-R buffer, pH 7	SWV	0.68 nM	0.68·10^−3^	NR	/	0.89–5.94 nM 5.94–44.6 nM	3.3123 µA nM^−1^ 0.5287 µA nM^−1^	NR	Real samples: tomato, apples, interferences: K^+^, Na^+^, Bi^3+^, SO_4_^2−^, NO_3_^−^, Cl^−^, fenitrothion	NR	[144]
Glyphosate	Hollow fibers-CuO-MWCNTs-IL	0.1 M phosphate buffer, pH 7	DPV	1.3 nM	1.30·10^−3^	4.3 nM	4.30·10^−3^	5 nM–1.1 µM	10.256 µA µM^−1^	NR	Real sample: river water, soil, interferences: Zn^2+^, Cd^2+^, Ca^2+^, Mg^2+^, Na^+^, NH_4_^+^, Br^−^, NO_3_^−^, SO_4_^2−^, PO_4_^3−^, glufosinate, bialaphos, tridemorph, chlorpyrifos, cypermethrin, (aminomethyl) phosphonic acid	92.19–103.25 (at 30–90 nM)	[145]
CBM simultaneously with yellow 50, tryptophan and caffeine	Pd NPs	0.1 M H_2_SO_4_	SWV	1.8·10^−8^	1.8·10^−2^	NR	/	0.2–1.6 µM	173 µA µM^−1^	6.9 (at 5.0·10^−7^ M)	Real samples: synthetic urine, river water, interferences: ascorbic acid, urea, NaCl, catechol, hydroquinone, Pd^2+^, Cd^2+^, uric acid, ranitidine, captopril	92.0–104 (at 2.5·10^−7^–5.0·10^−7^ M)	[146]
**Gold-Based Electrodes**													
MP	Au atomic clusters	0.1 M KCl	SWV	0.65 nM	0.65·10^−3^	NR	/	1–10 nM 10–80 µM	0.1468 µA nM^−1^ 1.8153 µA µM^−1^	2.5 (NR)	Real sample: water from bore wells, interferences: Cl^−^, NO_3_^−^, PO_4_^3−^, nitrophenol, nitrobenzene, nitroaniline	97 (at 10 nM)	[147]
Glyphosate	MIP chitosan	[Fe(CN)_6_]^3−/4−^, PBS	EIS	0.005 pg mL^−1^	2.96·10^−8^	NR	/	0.31 pg/mL–50 ng/mL	0.087 fg^−1^ mL	NR	Real samples: river water, interferences: gluphosinate-ammonium, chlorpyrifos, phosmet	NR	[153]
Methomyl	Au NP-Fe_3_O_4_ NP-chitosan	0.1 M B-R buffer, pH 6.9	amperometry	2.08·10^−5^ M	20.80	NR	/	2.97·10^−5^–3.47·10^−4^ M	0.03973 A M^−1^	NR	Real samples: lettuce, oilseed rape, spinach	90.02–98.26 (at 1.04·10^−3^ mol L^−1^)	[148]
Propamocarb	rGO	NR	CV	0.6 µM	0.60	NR	/	1–5 µM	101.1 µA µM^−1^ cm^−2^	NR	Real sample: cucumber, interferences: malathion, deltamethrin, cypermethrin	NR	[149]
**Glass-Based Electrodes**													
Diuron	PPy-ITO	0.1 M B-R buffer, pH 2.0	SWV	6.4·10^−7^ M	0.64	2.2·10^−6^ M	2.20	8.58·10^−7^–4.29·10^−5^ M	0.022 µA µM^−1^	NR	NR	NR	[150]
Diuron	PPy-MWCNT- ITO	0.1 M B-R buffer, pH 2.0	SWV	2.6·10^−7^ M	0.26	8.6·10^−7^ M	0.86	8.58·10^−7^–4.29·10^−5^ M	0.231 µA µM^−1^	NR	NR	NR	[150]
**Others**													
Glyphosate	Cu-BTC	0.1 M phosphate buffer, pH 5.5	DPV	1.4·10^−13^ M	1.4·10^−7^	NR	/	1.0·10^−12^–1.0·10^−9^ M 1.0·10^−9^–1.0·10^−5^ M	2.4767 µA M^−1^ 0.782 µA M^−1^	NR	Real sample: soybean, interferences: aminomethylphosphonic acid, Trichlorfon, CBM, Acetochlor, Thiram, K^+^, Ca^2+^, Zn^2+^, NO_3_^−^, Cl^−^, SO_4_^2^^−^	98.0–105.0 (at 0.10–1.00 µM)	[151]
MP	CaCO_3_-chitosan nanowall arrays	0.1 M phosphate buffer, pH 7.0	SWV	0.8 ng mL^−1^	3.04·10^−3^	NR	/	0.001–0.1 µg mL^−1^	591.8 µA mL µg^−1^	4.5 (at 0.1 µg mL^−1^)	Real sample: garlic, interferences: nitrobenzene, nitrophenol, PO_4_^2^^−^, SO_4_^3^^−^, NO_3_^−^	98.3–105.0 (at 0.002–0.050 µg mL^−1^)	[152]
Pirimiphos	CuO nanorods	0.25 M NaOH	CV	0.294 µM	2.94·10^−1^	NR	/	NR	2.833 µA mL ng^−1^	NR	Interferences: carbaryl, paraquat, sodium nitrate, sodium sulphate, toluene	NR	[154]
Paraoxon	CuO nanorods	0.25 M NaOH	CV	0.557 µM	5.57·10^−1^	NR	/	NR	1.657 µA mL ng^−1^	NR	Interferences: carbaryl, paraquat, sodium nitrate, sodium sulphate, toluene	NR	[154]
Parathion	CuO nanorods	0.25 M NaOH	CV	0.612 µM	6.12·10^−1^	NR	/	NR	1.425 µA mL ng^−1^	NR	Interferences: carbaryl, paraquat, sodium nitrate, sodium sulphate, toluene	NR	[154]
Chlorpyrifos	CuO nanorods	0.25 M NaOH	CV	0.571 µM	5.71·10^−1^	NR	/	NR	1.269 µA mL ng^−1^	NR	Interferences: carbaryl, paraquat, sodium nitrate, sodium sulphate, toluene	NR	[154]
CBM	BDD	0.1 M Na_2_HPO_4_, pH 2.0	SWV	1.2·10^−7^ M	1.2·10^−1^	4.0·10^−7^ M	4.0·10^−1^	0.5·10^−6^–15·10^−6^ M	0.08 A M^−1^	2.0 (5.0·10^−6^ M)	Real samples: pure and river water	90.0–96.0 (at 0.5·10^−6^ –40·10^−6^ M)	[155]
Fenamiphos	BDD	0.1 M Na_2_HPO_4_, pH 2.0	SWV	1.0·10^−7^ M	1.0·10^−1^	3.0·10^−7^ M	3.0·10^−1^	0.5·10^−6^–25·10^−6^ M	0.14 A M^−1^	3.1 (5.0·10^−6^ M)	Real samples: pure and river water	96.0–107.5 (at 0.5·10^−6^ –40·10^−6^ M)	[155]
CBM and fenamiphos (FNP) simultaneously	BDD	0.1 M Na_2_HPO_4_, pH 2.0	SWV	9.2 µg L^−1^ (CBM)	4.81·10^−2^ (CBM)	125 µg L^−1^ (CBM)	6.54·10^−1^ (CBM)	1·10^−6^–15·10^−6^ M (CBM) 0.5·10^−6^–7.0·10^−6^ M (FNP)	NR	NR	Real samples: pure and river water	NR	[155]
Carbaryl	Graphene-BDD	Acetate buffer, pH 5.6	CV	0.14 µM	0.14	0.46 µM	0.46	10–60 µM	1.85 µA µM^−1^ cm^−2^	NR	NR	NR	[156]
Carbaryl			DPV	0.07 µM	0.07	0.23 µM	0.23	1–12 µM	30.5 µA µM^−1^ cm^−2^	NR	NR	NR	
Paraquat			CV	0.01 µM	0.01	0.04 µM	0.04	0.2–1.2 µM	46.12 µA µM^−1^ cm^−2^	NR	NR	NR	
Paraquat			DPV	0.04 µM	0.04	0.13 µM	0.13	1–6 µM	30.8 µA µM^−1^ cm^−2^	NR	Real sample: fresh apple juice	NR	
Carbaryl (CBR) and paraquat (PQ) simultaneously			DPV	0.07 µM (CBR)	0.07	0.23 µM	0.23	1–6 µM	33.27 µA µM^−1^ cm^−2^	2.5 (8·10^−6^ M)	Real sample: fresh apple juice	NR	
				0.01 µM (PQ)	0.01	0.02 µM	0.02	0.2–1.2 µM	31.83 µA µM^−1^ cm^−2^	1.2 (1·10^−6^ M)			
Parathion	SiC NPs-MWCNTs–chitosan	0.2 M phosphate buffer, pH 6	DPV	20 ng mL^−1^	6.87·10^−2^	NR	/	50–2000 ng mL^−1^ 2000–10,000 ng mL^−1^	0.00198 µA ng^−1^ mL and 0.0006975 µA ng^−1^ mL	NR	Real samples: sweet potato leaf, Chinese cabbage, cucumber, interferences: NaNO_3_, MnSO_4_, CaCl_2_, citric acid, glucose, ascorbic acid	76.0–96.2 (at 1000–5000 ng mL^−1^)	[157]
CBM	CSS	0.1 M phosphate buffer, pH 7.0	DPV	4.7·10^−8^ M	4.7·10^−2^	NR	/	0.1–1.0 µM	0.18 A M^−1^	NR	Real samples: cabbages, apples, orange juice, interferences: chlorpyrifos, CBR, metomyl, atrazine, trifluralin, glyphosate, chloranil	96–101 (at 0.3–200.0 µM)	[158]
Diuron	PCNB	0.1 M phosphate buffer, pH 7.0	DPV	9.2·10^−7^ M	9.2·10^−1^	NR	/	1–10 µM	0.04 A M^−1^	NR	Real samples: cabbages, apples, orange juice, interferences: chlorpyrifos, CBR, metomyl, atrazine, trifluralin, glyphosate, chloranil	103–110 (at 2.0–796.0 µM)	[158]
Paraquat (PQ) and fenitrothion (FEN) simultaneously	Carbon ink	0.1 M phosphate buffer, pH 7.0	SWV	2.4·10^−8^ M (PQ) 6.4·10^−7^ M (FEN)	2.4·10^−2^ (PQ) 6.4·10^−1^ (FEN)	NR	/	0.1–1.0 µM (PQ) 1–10 µM (FEN)	2.47 A M^−1^ (PQ) 0.42 A M^−1^ (FEN)	NR	Real samples: cabbages, apples, orange juice, interferences: chlorpyrifos, CBR, metomyl, atrazine, trifluralin, glyphosate, chloranil	88.5–108 (at 0.2–7.96 µM, PQ) 93–107 (at 1.99–79.6 µM, FEN)	[158]
CBM	MWCNTs	0.04 M B-R buffer, pH 4.0	DPASV	0.049 µM	4.9·10^−2^	NR	/	0.25–2.50 µM	8.53 µA µM^−1^	12 (at 0.75 µM)	Real samples: mineral water, orange juice, interferences:	90–99 (at 0.8 µM)	[159]

## Abbreviations

ABIN	azobisisobutyronitrile
AC	activated carbon
AChE	Acetylcholinesterase
AC	atomic cluster
BDD	boron-doped diamond
BET	Brunauer–Emmett–Teller
BTC	benzene-1,3,5-tricarboxylic acid
B-R	Britton-Robinson
CBF	carbofuran
CBM	carbendazim
CBR	carbaryl
CMC	carboxymethyl cellulose
CMCh	carboxymethyl chitosan
CNH	carbon nanohorn
CNT	carbon nanotube
CPE	carbon paste electrode
CSS	carbon spherical shells
CTAB	cetyltrimethylammonium bromide
DMF	dimethylformamide
DPASV	differential pulse anodic stripping voltammetry
DPSV	differential pulse stripping voltammetry
DPV	differential pulse voltammetry
EGMRA	ethylene glycol maleic rosinate acrylate
EIS	electrochemical impedance spectroscopy
FEN	fenitrothion
FNM	fenamiphos
FS	fumed silica
GC	gas chromatography
GCE	glassy carbon electrode
GC-MS	gas chromatography-mass spectrometry
GNS	graphene nanosheets
GO	graphene oxide
g-C_3_N_4_	graphitic carbon nitride
HCNT	hydroxylated multiwall carbon nanotubes
HF	hollow fibre
HPLC	high-performance liquid chromatography
HS	headspace
IF	imprinting factor
IL	ionic liquid
ISO	isoproturon
ITO	indium tin oxide
LC	liquid chromatography
LOD	limit of detection
LOQ	limit of quantification
LPME	Liquid-phase microextraction
LSV	linear sweep voltammetry
MAA	methyl acrylic acid
MIL(Fe)	the iron-carboxylate nano metal-organic framework
MIP	molecularly imprinted polymer
MNP	magnetic nanoparticles
MP	methyl parathion
MUA	11-mercaptoundecanoic acid
MWCNT	Multi-wall carbon nanotubes
MWCNTPE	Multi-wall carbon nanotube paste electrode
NIP	Non-imprinted polymer
NP	nanoparticle
NPG	nanoporous gold
NR	not reported
NS	nanosheets
NT	nanotubes
NW	nanowires
OP	organophosphorous pesticides
PANI	polyaniline
PCL	poly(ε-caprolactone)
PCNB	Printex carbon nanoballs
PDA	polydopamine
PET	polyethylene terephthalate
POT	potentiometry
PPy	polypyrrole
PTH	polythiophene
QD	quantum dot
rGO	reduced graphene oxide
*R*	resistance
*R*_ct_	charge transfer resistance
SCE	saturated calomel electrode
SEM	scanning electron microscope
SPCE	screen-printed carbon electrode
SPE	screen-printed electrode
SPME	solid-phase microextraction
SS	stainless steel
SWASV	anodic stripping square-wave voltammetry
SWCNT	single-wall carbon nanotubes
SWV	square-wave voltammetry
*S*_BET_	specific surface area calculated using the BET method
TBOZ	zirconium n-butoxide
TEM	transmission electron microscope
TEOS	tetraethoxysilane
UiO	66-metal-organic framework ([Zr_6_O_4_(OH)_4_] clusters with 1,4-benzodicarboxylic acid struts)
4,4′-DDT	dichlorodiphenyltrichloroethane
